# Short-term efficacy of non-pharmacological interventions for global population with elevated blood pressure: A network meta-analysis

**DOI:** 10.3389/fpubh.2022.1051581

**Published:** 2023-01-13

**Authors:** Taihang Shao, Leyi Liang, Chengchao Zhou, Yaqian Tang, Wenqing Gao, Yusi Tu, Yue Yin, Daniel C. Malone, Wenxi Tang

**Affiliations:** ^1^Center for Pharmacoeconomics and Outcomes Research, China Pharmaceutical University, Nanjing, China; ^2^Centre for Health Management and Policy Research, School of Public Health, Cheeloo College of Medicine, Shandong University, Jinan, China; ^3^Department of Pharmacotherapy, College of Pharmacy, University of Utah, Salt Lake City, UT, United States; ^4^Department of Public Affairs Management, School of International Pharmaceutical Business, China Pharmaceutical University, Nanjing, China

**Keywords:** Bayesian network meta-analysis, prehypertension, non-pharmacological intervention, chronic disease management, blood pressure reduction

## Abstract

**Background:**

This study aims to compare the potential short-term effects of non-pharmacological interventions (NPIs) on prehypertensive people, and provide evidence for intervention models with potential in future community-based management.

**Methods:**

In this Bayesian network meta-analysis, Pubmed, Embase, and Web of science were screened up to 16 October 2021. Prehypertensive patients (systolic blood pressure, SBP 120–139 mmHg/diastolic blood pressure, DBP 80–89 mmHg) with a follow-up period longer than 4 weeks were targeted. Sixteen NPIs were identified during the scope review and categorized into five groups. Reduction in SBP and DBP was selected as outcome variables and the effect sizes were compared using consistency models among interventions and intervention groups. Grade approach was used to assess the certainty of evidence.

**Results:**

Thirty-nine studies with 8,279 participants were included. For SBP, strengthen exercises were the most advantageous intervention group when compared with usual care (mean difference = −6.02 mmHg, 95% CI −8.16 to −3.87), and combination exercise, isometric exercise, and aerobic exercise were the three most effective specific interventions. For DBP, relaxation was the most advantageous intervention group when compared with usual care (mean difference = −4.99 mmHg, 95% CI −7.03 to −2.96), and acupuncture, meditation, and combination exercise were the three most effective specific interventions. No inconsistency was found between indirect and direct evidence. However, heterogeneity was detected in some studies.

**Conclusion:**

NPIs can bring short-term BP reduction benefits for prehypertensive patients, especially exercise and relaxation. NPIs could potentially be included in community-based disease management for prehypertensive population once long-term real-world effectiveness and cost-effectiveness are proven.

**Systematic review registration:**

https://www.crd.york.ac.uk/PROSPERO/display_record.php?RecordID=151518, identifier: CRD42020151518.

## Introduction

Hypertension is one of the leading risk factors for morbidity and mortality around the world, which affect approximately one billion people (1–3). Elevated blood pressure, called prehypertension, defined as a blood pressure of 120–139/80–89 mmHg, is common and affects 25–50% of adults all over the world (4–6). Prehypertension confers a high risk of progression to hypertension and increases the risk of cardiovascular diseases with a 5-year progression rate of 40% (4, 7, 8). High prevalence and risk of prehypertension call for effective and cost-effective interventions. Current anti-hypertensive treatments include drug interventions and non-pharmacological interventions (NPIs) (9, 10), but previous research points out that there is a lack of evidence that early medication can bring benefits to prehypertensive people with no cardiovascular risk (5, 6), less to mention the unnecessary harm and economic loss brought by the resistance and side effects of anti-hypertensive drugs (11, 12). On the contrary, current available NPIs including exercises (e.g., aerobic exercise, resistance exercise), dietary intervention (e.g., salt reduction, alcohol reduction), relaxation (e.g., acupuncture, yoga), and so on Verma et al. (13), may be equally effective. Since NPIs focus more on changes in patient's behavior and lifestyle, they have no side effects and may be with potential cost-effectiveness (14). NPIs have been recommended by recent guidelines, which can be considered a priority in treating and managing prehypertensive people (6). Quite a few studies have proved that intensive lifestyle intervention as well as other NPIs can reduce the blood pressure (BP) of hypertensive and prehypertensive people in a short term (4, 15–18). However, the relative effects of NPIs are still unknown.

Although prehypertension has a large prevalence worldwide (6, 19), but patients are not managed in most countries currently. Simply using drug interventions may not be suitable to manage such a huge population since the economic burden can be large. NPIs are widely available and with low costs, can be good choices to be applied in management (20). However, implementing NPIs in current community-based chronic disease management still faced some barriers (21). NPIs require community-based chronic disease-management staff to have a medical background and additional professional training, and delivering NPIs is highly dependent on provider services (14, 22–24). However, these staffs are scarce, especially in many low- and middle-income countries (25). It is therefore necessary to explore the most effective and efficient type of service model which had the potential to be implemented. However, the evidence in this field is extremely lacking.

In this study, we aim to conduct a network meta-analysis to rank the short-term effects of NPIs among current available studies. The results will provide evidence for non-pharmacological treatment for prehypertensive patients from a global perspective, and hopefully lay a basis for the future inclusion of prehypertensive patients in community-based chronic disease management.

## Methods

We used a network meta-analysis to evaluate the short-term efficacy of NPIs on prehypertension. This research was reported following the Preferred Reporting Items for Systematic Reviews and Meta-Analyses (PRISMA) checklist (26). This research was registered with PROSPERO (registration number: CRD42020151518) and the protocol has been published (27).

### Patient and public involvement

Patients were not involved in this study.

### Literature search

We conducted a systematic search in PubMed, Web of Science, Embase, and the Cochrane Library up to 16 October 2021. We included randomized controlled trials and reasonably designed non-randomized controlled trials but excluded observational studies such as cross-sectional or cohort studies, systematic reviews and meta-analyses, and economic evaluations. We carried out a scope review and systematic review: the scope review was used to determine the NPIs included in the study, and the systematic review was then conducted to determine the studies included in the network meta-analysis. International guidelines were used to double-check the eligibility of interventions (6, 13, 28). The reference lists of relevant meta-analyses were scanned to identify other articles of interest. There was no limitation on the publication date of studies. The language of included studies was limited to English. Our search strategies and process are given in Supplementary material 1.

### Inclusion and exclusion criteria

All retrieved articles were imported into Noteexpress (3.2.0.7535, China Pharmaceutical University). Two independent researchers (THS and LYL) screened the literature for inclusion. Disagreements were discussed and consensus was reached in all cases. Literature was included in the systematic review if it met the following criteria.

#### Population

Study targets of adults aged >18 years whose BP status met the following two criteria were included: (1) diagnosed with prehypertension; (2) baseline BP between 120–139/80–89 mmHg. Subjects were excluded when they: (1) received antihypertensive agents; (2) had cardiovascular diseases (e.g., stroke, myocardial infarction); (3) had pregnancy-induced hypertension or pulmonary hypertension.

#### Intervention and control

Studies with at least one study arm using the following 15 NPIs were included: acupuncture, aerobic exercise, combination exercise, Dietary Approaches to Stop Hypertension (DASH), high Potassium, isometric exercise, lifestyle, meditation, normal exercise, alcohol reduction, resistance exercise, salt restriction, weight loss, yoga, and usual care. We merged these 15 interventions into five groups: relaxation, dietary intervention, strengthen exercise, lifestyle modification, and usual care, based on a comprehensive consideration of the type of interventions (exercise or dietary) and the intensity of intervention (strengthen or relax). The standardized descriptions of the interventions are shown in Table 1.

**Table 1:** Non-pharmacological interventions for prehypertensive patients.

**Name**	**Content**	**Intensity**	**Name**	**Content**	**Intensity**
Acupuncture	Including acupuncture and moxibustion. Acupuncture refers to acupuncture of certain acupoints by professionals, leaving the needle for 30 mins after getting qi. Moxibustion refers to the use of prefabricated moxibustion grass on certain acupoints on the body's surface, burning and ironing, using heat to stimulate the acupuncture points	Average 3 times/week, 30 min each time, lasting 6 weeks	Relaxation	Receive physical therapy by professionals. Or receive training by professionals, learn relaxing exercises, combine learning materials for independent exercises, including yoga, meditation	Average 4 times/week, 45 mins each time, lasting 12 weeks
Meditation	Transcendental meditation is aim to reduce stress. Participants are instructed by professional meditation instructors and then study and practice in groups to finish the mindfulness-based meditation program	Average 2 h/week, lasting 12 weeks			
Yoga	Guide by a professional yoga instructor to perform yoga exercises, including asanas, breathing and meditation	6–7 days a week, 45–60 min/d, lasting for 12 weeks			
Aerobic exercise	Use oxygen for energy, moderate to intensity exercise that lasts for a long time and has a rhythm, and the exercise intensity is 50–70% of the maximum heart rate. Including fast walking, jogging, cycling, mountain climbing and so on	3–4 times/week, 30–60 min/time, lasting 12 weeks	Strengthen exercise	Under the guidance of professionals, perform aerobic exercise (such as jogging, brisk walking, cycling, etc.), resistance exercise, isometric exercise or a combination of multiple forms of exercise at different target intensities	Average 4 times/week, 45 mins each time, lasting 8 weeks
Resistance exercise	Complete the specified actions according to the requirements and the purpose of the action design is to exercise the main muscle groups of the body. Totally seven actions, each action is repeated 10–15 times as a group, do 2–3 groups. Elastic ropes and other props are used under the supervision of the professionals	1 group of 12 reps; total 2 groups lasting 45 min; 3 times/week; lasting 6 months			
Normal exercise	Stretching or walking briskly for 30–60 mins under the supervision of professionals, the exercise intensity is about 5 km/h	45 mins/time; 3 times/week; lasting 8 weeks			
Isometric exercise	Use isometric handle equipment to exercise at the intensity of 20–30% MVC	10 min/d; every day; lasting 6 weeks			
Combination exercise	A comprehensive exercise program that combines endurance exercise, isometric exercise and aerobic exercise	3 times/week, an average of 50 min each time; lasting 7 weeks			
Salt reduction	Provide a measuring spoon, limit salt intake (maximum 3 g/day), or use alternative salt	≤ 3 g/day	Dietary	Reasonable diet, reduce sodium intake, reduce fat intake, reduce alcohol, increase potassium intake or adopt DASH diet	Adhere to certain diet every day. Conduct education every certain times
DASH	Emphasize a comprehensive diet, increase the intake of fruits, vegetables, whole grains and low-fat dairy products, and limit the intake of sodium, saturated fat and total fat	Eat according to DASH every day			
Alcohol reduction	Participants reduce their alcohol intake by substitute their drink to low-alcohol beer (0.9% v/v). Researchers provide 24 × 375 ml every 2 weeks	0.9% alcohol by volume drink 24 × 375 ml every 2 weeks			
High potassium	Participants either receive a salt substitute (potassium chloride) or receive dietary education by investigator to reduce sodium intake and increase potassium intake	Reduce sodium intake and increase potassium intake every day; education is conducted once a week			
Lifestyle	Give lifestyle suggestions, such as reasonable diet, reducing fat intake, proper exercise, weight control, reducing sodium intake, quitting smoking and alcohol, reducing mental stress. Then participants are required to change lifestyle according to these suggestions	Everyday	Lifestyle	Participants are required to change lifestyle or lose weight according to education	Everyday
Weight loss	Participants use the physical exercise plus low-calorie diet as well as specific nutrition intake for weight loss which are designed by researchers to achieve weight and BMI target	Dietary: every day. Physical exercise is recommended 30–45 min/d, 4–5 per week			
Usual care	No intervention, keep the original lifestyle habits unchanged	Everyday	Usual care	Regular blood pressure monitoring and health education is conducted without specific intervention	Follow up once a month and conduct health education once every 6 months

min, minute; d, day; MVC, maximum voluntary contraction; v/v, volume per volume; BMI, body mass index; DASH, dietary approaches to stop hypertension.

Left three columns represented the standardized description of 15 available non-pharmacological interventions for prehypertensive patients; Right three columns represented the standardized description of five non-pharmacological intervention groups (according to previous 15 specific interventions).

#### Outcome indicators

The main outcome indicators were changes in SBP and DBP whose follow-up time was no more than a year. We used mean differences instead of median differences as the effect size. The follow-up period of included studies did not exceed 1 year, the risk of cardiovascular events was therefore not reported. In addition, adverse events were not reported in most studies.

### Data extraction

Study characteristics extracted by four researchers (THS, LYL, YQT, and WQG) were as follows: title, first author, publication date, randomization, baseline characteristics (age, sex, country, number of participants, and lost to follow-up), details of interventions, follow-up time, baseline value as well as the changes of SBP and DBP after intervention.

### Risk of bias and evidence quality assessment

Two investigators (YST and YY) used the Cochrane Risk of Bias Tool 1.0 to evaluate the following items: random sequence generation, allocation concealment, blinding of outcome assessors, completeness of outcome data, selective outcome reporting, and other potential biases (29, 30). All six aspects would be evaluated as (1) low risk of bias, (2) unknown risk of bias, and (3) high risk of bias; a high-quality study should include more than four aspects with low risk of bias. However, blinding and allocation concealment would be difficult to achieve in NPIs. Therefore, we would make particular note of articles that did not involve blinding and allocation concealment but had valuable data (27).

We assessed the certainty of evidence using the grading of recommendations assessment, development and evaluation (GRADE) approach for network meta-analysis (31–35). Two people (THS and LYL) with experience in using GRADE rated each domain for each comparison separately and resolved discrepancies by consensus. We rated the certainty for each comparison and outcome as high, moderate, low, or very low, based on considerations of risk of bias, inconsistency, indirectness, publication bias, intransitivity, incoherence (difference between direct and indirect effects), and imprecision. Judgments of imprecision were made using a minimally contextualized approach, with a null effect as the threshold of importance (36). A recommended four-step approach was used in this study (35). In the first step, the effect sizes and confidence intervals of the direct evidence, indirect evidence, and network meta-analysis evidence were presented separately. In the second step, the quality of the direct evidence for each comparison group was graded without considering the imprecision. If the direct evidence was graded “high” and the contribution to the network meta-analysis results was greater than or equal to indirect evidence, no indirect evidence quality grading was required. The network meta-analysis evidence quality was directly assessed based on the direct evidence quality. Otherwise, indirect evidence quality grading was required. In the third step, based on the quality of direct evidence in the first-order loop of indirect evidence, the quality of indirect evidence was determined. The intransitivity should also be considered. In the fourth step, based on the level of direct evidence and/or indirect evidence, and considering inconsistency and imprecision, the quality of evidence for network meta-analysis was finalized and presented.

### Statistical analysis

We carried out a network meta-analysis using the Bayesian framework with the same priors for the variance and effect parameters. A plausible prior for the variance parameter and a uniform prior for the effect parameter suggested in a previous study based on empirical data were used in this network meta-analysis (37). We calculated the mean difference (MD) as the effect size using the reported means and standard deviations (SD) of changes in SBP and DBP. If the original study reported the standard error (SE), we would convert it to the SD through the sample size (*n*):


$$\begin{array}{l} SE=\frac{SD}{\sqrt{n}} \end{array}$$

If the changes were not reported in the article but the BP at the start and end of the follow-up period were reported, we calculated the mean and SD using the following formula recommended in the Cochrane Handbook (29):


$$\begin{array}{l} mean_{change}=mean_{final}-mean_{baseline}\\ \;SD_{change}=\sqrt{SD_{baseline}^{2}+SD_{final}^{2}-2^{*}Corr^{*}SD_{baseline}^{*}SD_{final}} \end{array}$$

The two most widely used models in network meta-analysis were the fixed effect model and the random effects model (38). The fixed effect model was built under the assumption of existing no heterogeneity. But this assumption was recognized to be unrealistic. If the fixed effect model was applied when heterogeneity existed, uncertainty intervals become artificially narrow. Therefore, the random effects model was preferred since it assumed and accounted for unexplained heterogeneity. In this network meta-analysis, we used a random effects model as the most appropriate and conservative method to explain the heterogeneity among the included studies (38, 39). We used a Markov chain Monte Carlo simulation with four chains with scattered initial values, a total of 50,000 iterations, and annealed after 5,000 iterations. The convergence of the model was judged by the Brooks–Gelman–Rubin method (40).

A ranking probability curve of each treatment was provided by calculating the probability of each arm to achieve the best rank among all. We judged the inconsistency by comparing the deviance information criterion (DIC) between the consistency and inconsistency models (41). Evaluating local incoherence between the direct and indirect comparisons, and obtaining indirect estimation was done by the node-splitting models (42). We calculated the Bayesian *P* value to estimate the measure of the conflict between direct and indirect evidence (43). The heterogeneity between studies was determined by heterogeneity analysis (including unrelated study effects model, unrelated mean effects model, and consistency model), and Q test and *I*^2^ statistic were used to reflect the heterogeneity (*I*^2^higher than 75% was considered with high heterogeneity; smaller than 25% was considered with low heterogeneity) (44).

All statistical tests were conducted as two-sided, and a *P* < 0.05 was considered as being statistically significant. The network meta-analysis was performed using R software (https://www.r-project.org).

## Results

### Study selection

**Figure 1:**
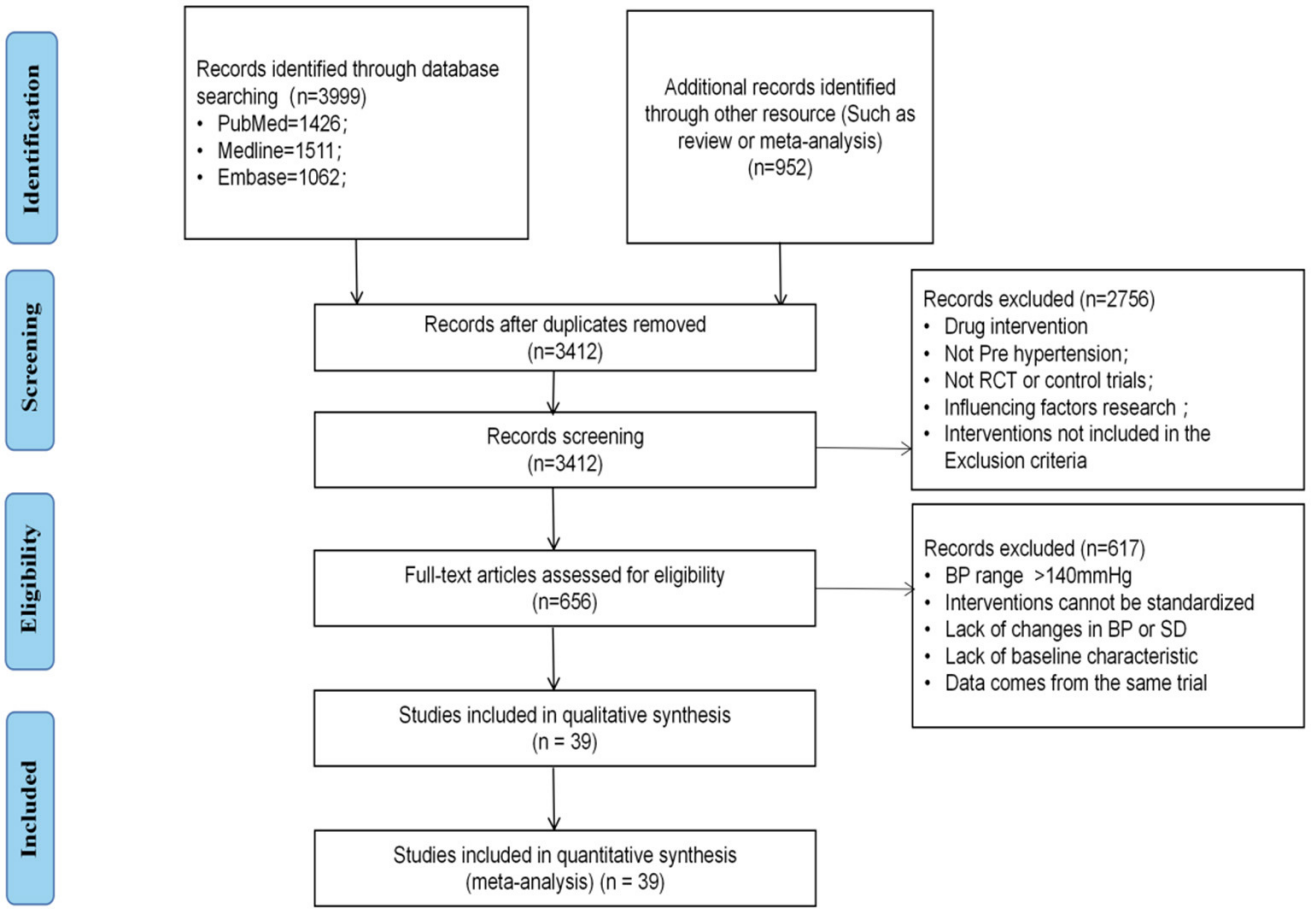
Flow chart of literature search and article inclusion. RCT, randomized controlled trials; BP, blood pressure; SD, standard difference.

Our search identified a total of 4,951 references. After duplication, 3,412 studies underwent further analysis, of which 2,756 were excluded after reading the title or abstract and a further 617 were excluded after reading the full text. The remaining 39 studies involved 15 interventions and 8,279 patients were included in the analysis (45–83). The study flow chart is shown in Figure 1. The baseline patient characteristics are shown in the Supplementary material 2.

### Network meta-analysis

**Figure 2:**
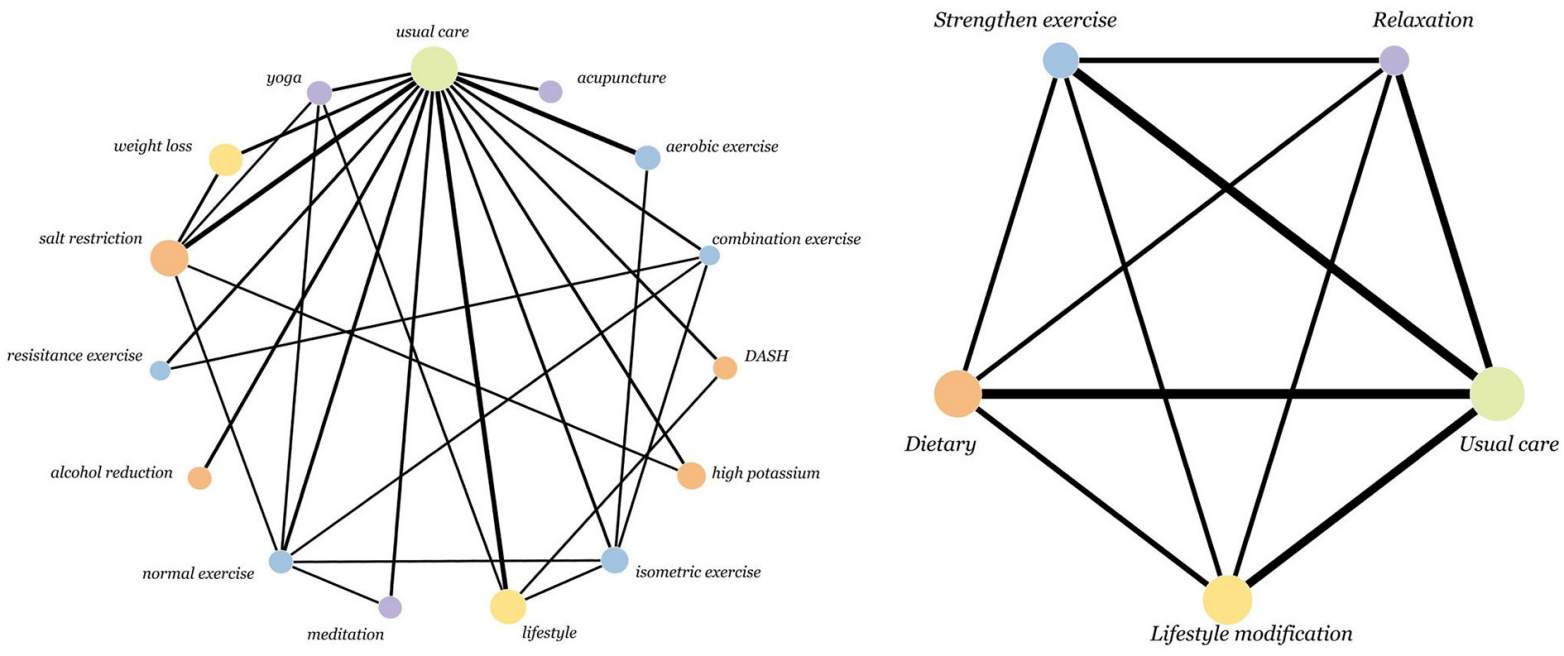
Network of intervention treatments included in meta-analysis. The size of the nodes represents the sample size. The thickness of the lines represents the number of studies included in the comparison.

The network evidence plots for SBP and DBP were the same (as shown in Figure 2).

**Figure 3:**
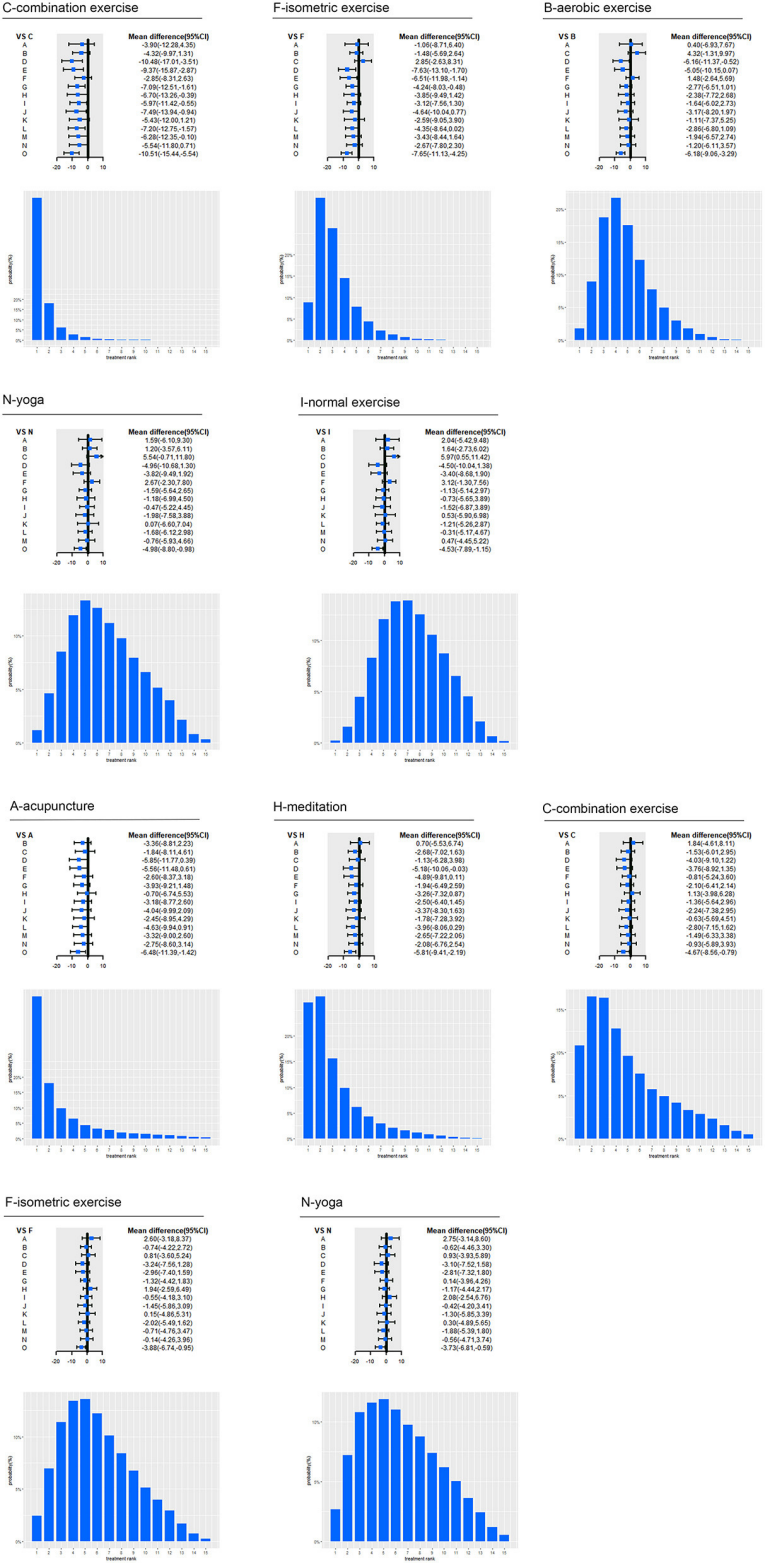
Effect of top five interventions on SBP and DBP. A, acupuncture; B, aerobic exercise; C, combination exercise; D, DASH; E, high Potassium; F, isometric exercise; G, lifestyle; H, meditation; I, normal exercise; J, reduced alcohol; K, resistance exercise; L, salt restriction; M, weight loss; N, yoga; O, usual care; CI, confidence interval; SBP, systolic blood pressure; DBP, diastolic blood pressure. Forest plot represents the relative effects of the other interventions with one intervention as a reference (mean difference, 95% CI, mmHg); Bar plot represents the probability of ranking of the reference intervention.

Among the included intervention strategies, combination exercise (69.71%) ranked first in the reduction of SBP, followed by isometric exercise (33.30%), aerobic exercise (18.75%), yoga (11.90%) and normal exercise (12.08%). And acupuncture (46.09%) ranked first in the reduction of DBP, followed by meditation (27.67%), combination exercise (16.39%), isometric exercise (13.42%), yoga (11.86%) (as shown in Figure 3).

Specifically, for SBP, aerobic exercise (−6.18 mmHg, 95% CI −9.06 to −3.29; moderate certainty), combination exercise (−10.51 mmHg, −15.44 to −5.54; moderate certainty), isometric exercise (−7.65 mmHg, −11.13 to −4.25; moderate certainty), lifestyle (−3.4 mmHg, −5.89 to −0.94; moderate certainty), normal exercise (−4.53 mmHg, −7.89 to −1.15; low certainty), salt restriction (−3.3 mmHg, −6.02 to −0.7; moderate certainty), weight loss (−4.23 mmHg, −7.97 to −0.58; moderate certainty) and yoga (−4.98 mmHg, −8.8 to −0.98; low certainty) significantly lowered SBP compared with usual care. Aerobic exercise (−6.16 mmHg, −11.37 to −0.52; moderate certainty) had a significant SBP reduction compared with DASH. Combination exercise had a significant SBP reduction compared with DASH (−10.48 mmHg, −17.01 to −3.51; moderate certainty), high potassium (−9.37 mmHg, −15.87 to −2.87; moderate certainty), lifestyle (−7.09 mmHg, −12.51 to −1.61; moderate certainty), meditation (−6.7 mmHg, −13.26 to −0.39; moderate certainty), normal exercise (−5.97 mmHg, −11.42 to −0.55; low certainty), alcohol reduction (−7.49 mmHg, −13.94 to −0.94; moderate certainty), salt restriction (−7.2 mmHg, −12.75 to −1.57; moderate certainty) and weight loss (−6.28 mmHg, −12.35 to −0.1; moderate certainty). Isometric exercise significantly lowered SBP compared with lifestyle (−4.24 mmHg, −8.03 to −0.48; very low certainty), DASH (−7.63 mmHg, −13.1 to −1.7; low certainty), and high Potassium (−6.51 mmHg, −11.98 to −1.14; low certainty). For DBP, acupuncture (−6.48 mmHg, −11.39 to −1.42; moderate certainty), aerobic exercise (−3.12 mmHg, −5.51 to −0.73; moderate certainty), combination exercise (−4.67 mmHg, −8.56 to −0.79; moderate certainty), isometric exercise (−3.88 mmHg, −6.74 to −0.95; low certainty), lifestyle (−2.56 mmHg, −4.56 to −0.58; moderate certainty), meditation (−5.81 mmHg, −9.41 to −2.19; moderate certainty), normal exercise (−3.32 mmHg, −6.05 to −0.59; low certainty), weight loss (−3.16 mmHg, −6.14 to −0.29; moderate certainty) and yoga (−3.73 mmHg, −6.81 to −0.59; moderate certainty) significantly lowered BP compared with usual care. Meditation (−5.18 mmHg, −10.06 to −0.03; moderate certainty) had a significant BP reduction than DASH.

Among the categorized intervention groups, Strengthen exercise (72.54%) ranked first in the reduction of SBP, followed by relaxation (54.56%), lifestyle modification (59.6%), dietary (71.62%), and usual care (99.08%). And relaxation (87.66%) ranked first in the reduction of DBP, followed by Strengthen Exercise (61.93%), lifestyle modification (60.55%), dietary (82.34%), and usual care (98.73%).

**Figure 4:**
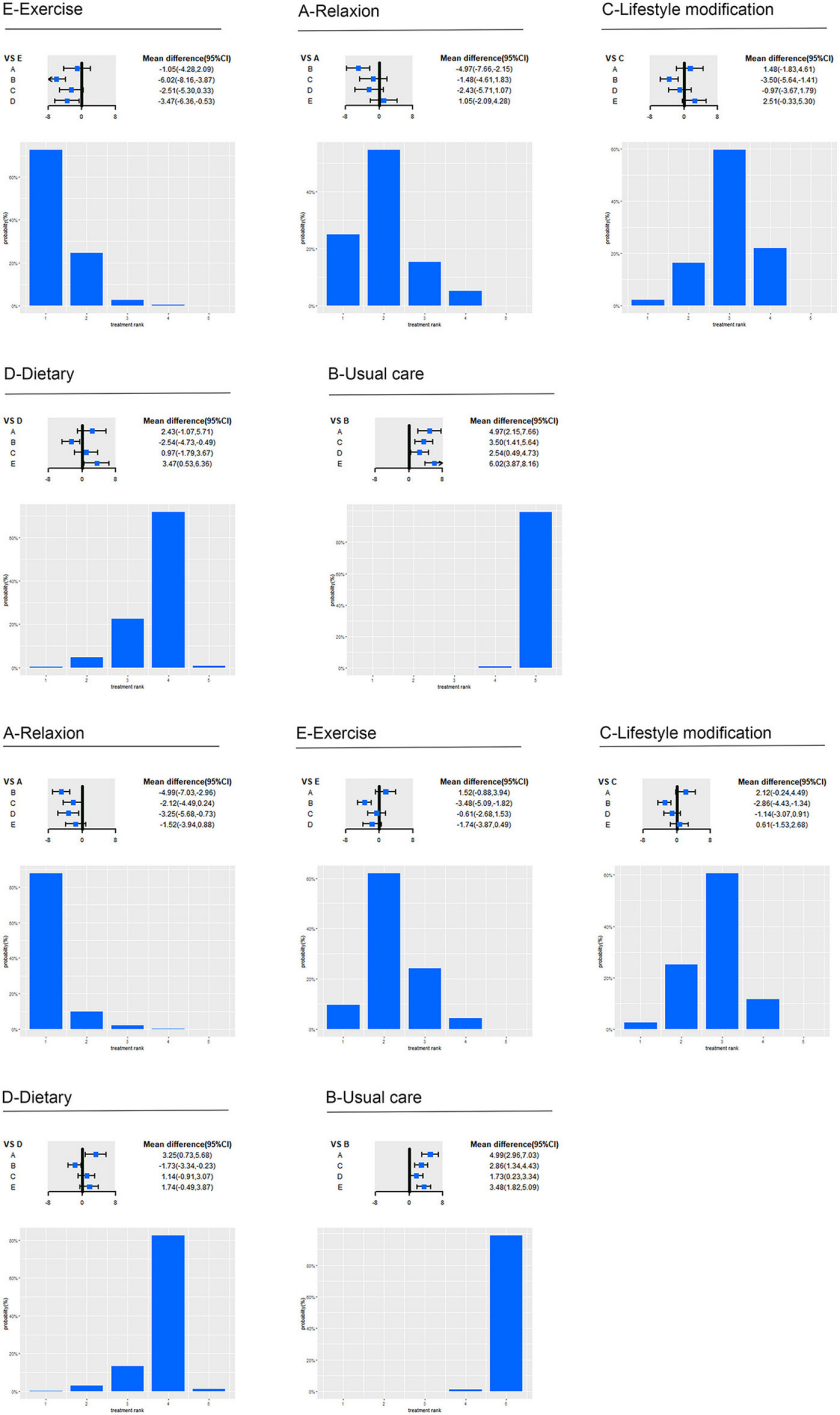
Effect of five intervention groups on SBP and DBP. A, relaxation; B, usual care; C, lifestyle; D, Dietary; E, strengthen exercise; CI, confidence interval; SBP, systolic blood pressure; DBP, diastolic blood pressure. Forest plot represents the relative effects of the other intervention groups with one intervention group as a reference (mean difference, 95% CI, mmHg). Bar plot represents the probability of ranking of the reference intervention group.

Specifically, for SBP (as shown in Figure 4), relaxation (−4.97 mmHg, −7.66 to −2.15; low certainty), lifestyle modification (−3.5 mmHg, −5.64 to −1.41; moderate certainty), dietary (−2.54 mmHg, −4.73 to −0.49; low certainty) and strengthen exercise (−6.02 mmHg, −8.16 to −3.87; low certainty) significantly reduced BP compared with usual care. Strengthen exercise (−3.47 mmHg, −6.36 to −0.53; moderate certainty) significantly reduced BP compared with dietary. For DBP, relaxation (−4.99 mmHg, −7.03 to −2.96; moderate certainty), lifestyle modification (−2.86 mmHg, −4.43 to −1.34; moderate certainty), dietary (−1.73 mmHg, −3.34 to −0.23; very low certainty) and strengthen exercise (−3.48 mmHg, −5.09 to −1.82; low certainty) significantly reduced BP compared with usual care. Relaxation (−3.25 mmHg, −5.68 to −0.73; moderate certainty) significantly reduced BP compared with dietary.

### Inconsistency and heterogeneity

Results of node-splitting and heterogeneity tests are shown in Supplementary material 3 for detail.

When compared among specific intervention programs, no inconsistency was found in SBP nor DBP, as indicated by the model parameters (consistency test for SBP and DBP were DIC = 162.70) and DIC = 164.43, respectively; inconsistency test for SBP and DBP were DIC = 166.97 and DIC = 167.49, respectively. The results of node-splitting analysis showed that there was no local incoherence in both of the models since every Bayesian *P* > 0.05.

When compared among intervention groups, no inconsistency was found in SBP nor DBP, as indicated by the model parameters (consistency test for SBP and DBP were DIC = 138.37 and DIC = 139.59, respectively; inconsistency test for SBP and DBP were DIC = 140.64 and DIC = 141.31, respectively). The results of node-splitting analysis showed that there was no local incoherence in both of the models since every Bayesian *P* > 0.05.

The heterogeneity test showed that there was high heterogeneity between some studies for both pair-wise pooled effects and consistency effects. Potential explanations are given in a later discussion.

### Risk of bias

The specific literature quality assessment diagram and risk of bias summary are shown in Supplementary material 4. After assessing the quality of the literature by the Cochrane Handbook, we found that 27 studies reported the implementation of randomization, 8 studies reported the allocation concealment, and 15 studies reported on the implementation of blinding. There was no selective reporting bias or result bias in all studies. Because these studies were aimed at NPIs, some study designs could not apply the blinding. In summary, the quality of the articles included in this network meta-analysis was moderate. Detailed results of the certainty evaluation of evidence are shown in Supplementary material 5. Overall, most evidence was concentrated in the moderate and low grades since the existence of the risk of bias, inconsistency, indirectness, intransitivity, and imprecision.

## Discussion

This study evaluated the short-term effects of 16 NPIs in patients with prehypertension. Considering the impact on community-based chronic disease-management staff and their need for professional cooperation (25), we merged 16 intervention items into five intervention groups. We evaluated the effects using a network meta-analysis with BP reduction as the outcome indicator and found that combination exercise, isometric exercise, aerobic exercise, yoga, and normal exercise were the top five in SBP reduction, and acupuncture, meditation, combination exercise, isometric exercise and yoga for DBP reduction. Also, according to our studies, sports intervention had more absolute SBP reduction and relaxation had more absolute DBP reduction than other interventions. Strengthen exercise and relaxation rank top two in both SBP and DBP reduction.

There were no inconsistencies but slight heterogeneity in this study. This was likely due to differences in the baseline characteristics (e.g., age, sex, and ethnicity) of the patients included in each study, as well as differences in the interventions adopted by each of the studies. Although the interventions share the same purpose, their contents were slightly different, given that current guidelines did not give standardized strategies for non-pharmacological intervention. Patient compliance and completion rates may also differ among studies. However, this type of heterogeneity was unavoidable.

Numerous studies have examined the short-term anti-hypertensive effects of NPIs. Williamson et al. (18) found that 3–6 months of exercise effectively reduced SBP (−4.40 mmHg, 95% CI −5.78 to −3.01) and DBP (−4.17 mmHg, 95% CI −5.42 to −2.93) in patients aged 18–40 years with both prehypertension and hypertension. Ndanuko et al. (84) conducted a meta-analysis on the BP-reducing effects of MBSR (Mindfulness-Based Stress Reduction) program in both prehypertensive and hypertensive patients and showed that MBSR reduced SBP by 6.64 mmHg and DBP by 2.47 mmHg. Khandekar et al. (85) conducted a meta-analysis of the anti-hypertensive effects of yoga and showed that both SBP (standard MD = −0.62, 95% CI −0.83 to −0.41) and DBP (standard MD = −0.81, 95% CI −1.39 to −0.22) were significantly reduced in the yoga group compared with the control group. According to Liao et al.'s study (86), massage significantly reduced SBP (−7.39 mmHg) and DBP (−5.04 mmHg) in people with both prehypertension and hypertension. Fu et al. (20) reported that the DASH diet was most effective for adults with prehypertension to established hypertension, followed by aerobic exercise and isometric training, which also had obvious effects on BP reduction. Population in current evidence are hypertensive patients or combined with prehypertensive people. Population in current evidence are hypertensive patients or combined with prehypertensive people. Pooled evidence studies target on the BP reduction effect of NPIs in prehypertensive people were lacking. In addition, current studies of the anti-hypertensive effects of NPIs have tended to target one specific intervention. So the current results filled a gap in proving and comparing the short-term effects of NPIs in people with prehypertension.

This was the first study to evaluate the short-term efficacy of NPIs in prehypertensive people. Our results not only supplemented existing evidence in this area but also had important implications for the management of chronic diseases in countries who had a high disease burden of hypertension but with limited medical resources and community-based chronic disease-management staff. Early prevention of hypertension through NPIs can be a potential way to reduce the disease burden. This meant that government administrators in these countries can start to initiate training programs that could reduce the BP of people with prehypertension effectively for community-based chronic disease-management staff to be prepared. For decision-makers, a comprehensive analysis of which types of interventions could be more effective will provide useful evidence to make the optimal health decision. In this study, strengthen exercise and relaxation, which could bring more short-term BP reduction than other interventions according to current evidence, may be considered the priority for government administrators and community-based chronic disease-management staff. Nevertheless, long-term effects of these NPIs with great short-term BP reduction benefits should be further examined (including the number of CVD events avoided), which can provide more evidence for decision-makers.

It is necessary to note that this study is not without shortcomings. First, the standardization of interventions in this study was carried out following the guidelines, still it may leave to subjectivity. Second, due to differences in population baseline of included studies, the heterogeneity could not be avoided. Therefore, in our certainty of the evidence analysis, most comparisons were downgraded in the indirectness of evidence due to the differences in population and intervention. Third, since individual patient data were not available, subgroup analyses were not conducted in this study. However, the heterogeneity caused by some key subgroups (e.g., baseline blood pressure, age) should not be ignored. Fourth, since long-term studies were lacking, only BP change could be selected as the outcome indicator in this study. Without outcome indicators like cardiovascular events or hypertension progression, long-term real-world effectiveness of NPIs could not be recognized. Even though we had proved that NPIs are effective for prehypertensive people, whether they are cost-effective was still unknown. For further research, more high-quality research with long-term outcome projections should be published to fill the gap in this field. Studies are needed to target people with different clinical characteristics. Empirical studies on the inputs and outputs of NPIs in prehypertensive people are also needed to explore the cost-effectiveness and feasibility of implementation in a specific region.

## Conclusion

To date, there is no systematic study revealing the comparative effects of NPIs for prehypertensive patients. Our study indicates that strengthening exercise (including combination exercise, isometric exercise and aerobic exercise) and relaxation (including acupuncture, meditation, and yoga) have potential to be educated and applied in community-based chronic disease management. This will provide evidence for countries who have a high disease burden of hypertension but with limited medical resources and staff to prevent or delay the disease progression from prehypertension to hypertension. However, to have a decision on whether prehypertensive patients should be regularly managed and which strategy to be considered, further studies on cost-effectiveness and affordability are needed.

## Data availability statement

The original contributions presented in the study are included in the article/Supplementary material, further inquiries can be directed to the corresponding authors.

## Author contributions

WT and TS designed the study and interpreted findings. TS and LL wrote initial drafts of the manuscript, developed the model, performed all model analyses, and visualized the data. TS, LL, YTa, WG, YTu, and YY conducted the literature search, screen, and extract the data. TS, LL, CZ, WT, and DM revised and polished the initial manuscript drafts. All authors reviewed the manuscript. All authors had full access to all the data in the study and the corresponding authors had final responsibility for the decision to submit for publication.

## Supplementary material

Supplementary material 1 Search strategies of this study. Including reverse search and forward search.

Supplementary material 2 Main characteristics of included trials.

Supplementary material 3 Results of node-splitting and heterogeneity test.

Supplementary material 4 “Quality assessment diagram” and “Risk of bias summary.”

Supplementary material 5 Detailed results of the certainty of the evidence analysis.
