# Supplementary material for: Short-term efficacy of non-pharmacological interventions for global population with elevated blood pressure: A network meta-analysis
Source: Front Public Health. 2023 Jan 13;10:1051581. doi: 10.3389/fpubh.2022.1051581 (PMC9880179; doi:10.3389/fpubh.2022.1051581)
Supplement: Supplementary material 1 — Search strategies of this study. Including reverse search and forward search. [file Table_1.DOCX]

**Supplemental material 1**

**Search Strategies**

**Reverse search strategy**

**(PubMed as example)**

This study conducted the reverse search first. The intervention measures were not restricted so as to search as much as possible. After obtaining the studies, sort the frequency of the keywords and text words, and eliminate the intervention items that appear less frequently or irrelevant. Finally a total of 46 keywords were retrieved as follows: *diet, DASH, Dietary pattern, low salt/sodium , Alcohol, *nutrition, potassium, Weight loss, Lifestyle, *Yoga, *Qigong, Tai Ji, exercise, training, *Meditation, Sleep duration, Sleep quality, *daytime sleepiness, *Afternoon napping, *health education, Health Promotion, Health Screening Program, *Health Services Accessibility, Patient Education, *Health Knowledge, Attitudes, Practice, *self-care/management, *self-management, Mental Health, Self Report, self management, Self Efficacy, *smartphone, wechat, smart devices, telehealth, Electronic health, *online health community, *ambulatory blood pressure monitoring, Telemedicine, online health, family doctor, multidisciplinary team, coordinated service/continuity of care, Medical union OR medical alliance OR integrated care , payment/incentives/reimbursement, delivery system.* Then we brought these keywords into the forward search strategies and searched in major databases.

Taking PubMed as an example, the reverse search strategy was as follows：*(((((((((pre hypertension[MeSH Terms]) OR (Hypertension[MeSH Terms])) OR (High normal blood pressure[MeSH Terms])) OR (pre hypertension[Title/Abstract])) OR (High normal blood pressure[Title/Abstract])) OR (High blood pressure[Title/Abstract])) NOT ((((((Masked hypertension[Title] OR Masked hypertension[Mesh Terms]) OR (white coat hypertension [Title] OR white coat hypertension [Mesh Terms])) OR (pregnancy [Title] OR Pregnancy Induced Hypertension [Mesh Terms])) OR (pulmonary [Title] OR Pulmonary Hypertension [Mesh Terms])) OR (Renal [Title] OR Renal Hypertension [Mesh Terms])) OR (Retinopathy [Title] OR Hypertensive Retinopathy [Mesh Terms])) NOT secondary hypertension[Title]) AND (((((((cohort studies[mesh:noexp]) OR (longitudinal studies[mesh:noexp])) OR (follow-up studies[mesh:noexp])) OR (prospective studies[mesh:noexp])) OR (retrospective studies[mesh:noexp])) OR (cohort[TIAB] OR longitudinal[TIAB] OR prospective[TIAB] OR retrospective[TIAB])) OR (((randomized controlled trial [Publication Type]) OR (controlled clinical trial [Publication Type])) OR (randomized [Title/Abstract] OR randomly [Title/Abstract] OR placebo [Title/Abstract] OR trial [Title/Abstract] OR groups[Title/Abstract])))) AND (China OR chinese)) NOT (((((((Influencing factors[Title]) OR (Mechanism[Title])) OR (Rat[Title])) OR (Pregnancy[Title])) OR (Mouse[Title])) OR (Animal[Title])) OR (Rabbit[Title]))*

(Searching cutoff Date: August 6, 2021)

**Forward search strategy**

(Searching cutoff Date: October 16, 2021)

**Pubmed：**

| **No.** | **Query** |
| --- | --- |
| #1 | Prehypertension[MeSH Terms] |
| #2 | Prehypertension[Title/Abstract] |
| #3 | High normal blood pressure[Title/Abstract] |
| #4 | High risk of hypertension[Title/Abstract] |
| #5 | Elevated blood pressure[Title/Abstract] |
| #6 | 120-139 / 80-89 mmHg[Title/Abstract] |
| #7 | 130-139 / 80-89 mmHg[Title/Abstract] |
| #8 | raised blood pressure[Title/Abstract] |
| #9 | #1 OR #2 OR #3 OR #4 OR #5 OR #6 OR #7 OR #8 |
| #10 | Masked hypertension[MeSH Terms] OR Masked hypertension[Title/Abstract] |
| #11 | secondary hypertension[Title/Abstract] |
| #12 | white coat hypertension[Title/Abstract] OR white coat hypertension[MeSH Terms] |
| #13 | pregnancy[Title/Abstract] OR Pregnancy Induced Hypertension[MeSH Terms] |
| #14 | Pulmonary[Title/Abstract] OR Pulmonary Hypertension[MeSH Terms] |
| #15 | Renal [Title/Abstract] OR Renal Hypertension [Mesh Terms] OR Retinopathy [Title/Abstract] OR Hypertensive Retinopathy [Mesh Terms] |
| #16 | Influencing factors[Title] OR risk factors[Title] |
| #17 | Mechanism[Title/Abstract] |
| #18 | protocol[Title] |
| #19 | rats [Title/Abstract] OR mouse [Title/Abstract] OR rabbits [Title/Abstract] OR dogs [Title/Abstract] |
| #20 | animals [MeSH Terms] OR humans [MeSH Terms] |
| #21 | #10 OR #11 OR #12 OR #13 OR #14 OR #15 OR #16 OR #17 OR #18 OR #19 OR #20 |
| #22 | Intervention[Title/Abstract] OR non-pharmacological interventions [All Fields] OR non-pharmaceutical interventions[All Fields] |
| #23 | Diet [All Fields] OR Dietary pattern [All Fields] OR DASH [All Fields] OR salt [All Fields] OR sodium [All Fields] OR alcohol [All Fields] OR cigarette [All Fields] OR tobacco [All Fields] OR smoking [All Fields] OR nutrition [All Fields] OR potassium [All Fields] |
| #24 | Lifestyle [All Fields] OR exercise [All Fields] OR training [All Fields] OR Weight loss [All Fields] OR Yoga [All Fields] OR Qigong [All Fields] OR Tai Ji [All Fields] OR tai chi [All Fields] OR Meditation [All Fields] |
| #25 | Sleep duration [All Fields] OR Sleep quality [All Fields] OR daytime sleepiness [All Fields] OR Afternoon napping[All Fields] |
| #26 | Health education [All Fields] OR Health Promotion [All Fields] OR Health Screening Program [All Fields] OR Health Services Accessibility [All Fields] OR Health Services Accessibility [All Fields] OR Patient Education [All Fields] OR Knowledge Attitude/Belief [All Fields] and Practice [All Fields] OR KAP [All Fields] OR Health Knowledge, Attitudes, Practice[All Fields] |
| #27 | self-care [All Fields] OR self-management [All Fields] OR Acupuncture [All Fields] OR Mental Health [All Fields] OR Self Report [All Fields] OR Self Efficacy [All Fields] |
| #28 | wearable devices [All Fields] OR smartphone [All Fields] OR wechat [All Fields] OR smart devices [All Fields] OR telehealth [All Fields] OR Electronic health [All Fields] OR online health community [All Fields] OR ambulatory blood pressure monitoring [All Fields] OR Telemedicine [All Fields] OR online health [All Fields] |
| #29 | family doctor [All Fields] OR multidisciplinary team [All Fields] OR coordinated service [All Fields] OR continuity of care [All Fields] OR Medical union [All Fields] OR medical alliance [All Fields] OR integrated care [All Fields] |
| #30 | payment [All Fields] OR incentives [All Fields] OR reimbursement [All Fields] OR delivery system [All Fields] |
| #31 | #22 OR #23 OR #24 OR #25 OR #26 OR #27 OR #28 OR #29 OR #30 |
| #32 | randomized controlled trial [Publication Type] |
| #33 | controlled clinical trial [Publication Type] |
| #34 | randomized [Title/Abstract] OR randomly [Title/Abstract] OR placebo [Title/Abstract] OR trial [Title/Abstract] OR groups[Title/Abstract] |
| #35 | #32 OR #33 OR #34 |
| #36 | #9NOT #21 AND #31 AND #35 |

**Embase**

| **No.** | **Query** |
| --- | --- |
| #1 | 'prehypertension'/exp |
| #2 | 'prehypertension':ti,ab,kw |
| #3 | 'high normal blood pressure':ti,ab,kw |
| #4 | high risk of hypertension':ti,ab,kw |
| #5 | elevated hypertension':ti,ab,kw |
| #6 | 120-139 / 80-89 mmhg':ti,ab,kw |
| #7 | 130-139 / 80-89 mmhg':ti,ab,kw |
| #8 | raised blood pressure':ti,ab,kw |
| #9 | #1 OR #2 OR #3 OR #4 OR #5 OR #6 OR #7 OR #8 |
| #10 | 'masked hypertension'/exp OR 'masked hypertension':ti,ab,kw |
| #11 | 'white coat hypertension'/exp OR 'white coat hypertension':ti,ab,kw |
| #12 | 'secondary hypertension':ti,ab,kw |
| #13 | 'maternal hypertension'/exp OR 'maternal hypertension':ti,ab,kw OR 'pregnancy':ti,ab,kw |
| #14 | 'pulmonary hypertension'/exp OR 'pulmonary':ti,ab,kw |
| #15 | 'renovascular hypertension'/exp OR 'renovascular hypertension' OR 'renal':ti,ab,kw OR 'renovascular hypertension':ti,ab,kw |
| #16 | 'hypertension retinopathy'/exp OR 'retinopathy':ti,ab,kw |
| #17 | 'influencing factors':ti OR 'mechanism':ti OR 'risk factor':ti |
| #18 | 'protocol':ti |
| #19 | 'animal':ti,ab,kw OR 'rat':ti,ab,kw OR 'mouse':ti,ab,kw OR 'rabbit':ti,ab,kw OR 'dog':ti,ab,kw |
| #20 | 'animal'/exp NOT 'human'/exp |
| #21 | #10 OR #11 OR #12 OR #13 OR #14 OR #15 OR #16 OR #17 OR #18 OR #19 OR #20 |
| #22 | #9 NOT #21 |
| #23 | diet OR 'dietary pattern' OR dash OR 'salt' OR 'sodium' OR alcohol OR cigarette OR tobacco OR smoking OR nutrition OR potassium |
| #24 | 'lifestyle' OR 'exercise' OR 'training' OR 'weight loss' OR 'yoga' OR 'qigong' OR 'tai chi' OR 'tai ji' OR 'meditation' |
| #25 | 'sleep time' OR 'sleep duration' OR 'sleep quality' OR 'afternoon napping' OR 'daytime sleepiness' |
| #26 | 'health education' OR 'health promotion' OR 'health screening program' OR 'health care access' OR 'patient education' OR 'knowledge, attitudes, practice' OR 'kap' OR 'attitude' OR 'health services accessibility' OR 'knowledge attitude/belief and practice' |
| #27 | 'self care' OR 'self management' OR 'acupuncture' OR 'mental health' OR 'self report' OR 'self efficacy' |
| #28 | 'wearable device' OR 'smartphone' OR 'wechat' OR 'smart device' OR 'telehealth' OR 'electronic health' OR 'online health community' OR 'ambulatory blood pressure monitoring' OR 'telemedicine' OR 'online health' OR 'wearable devices' OR 'social media' OR 'smart devices' |
| #29 | 'family doctor' OR 'multidisciplinary team' OR 'coordinated service' OR 'continuity of care' OR 'medical union' OR 'medical alliance' OR 'integrated care' |
| #30 | payment' OR 'incentive' OR 'reimbursement' OR 'delivery system' OR incentives |
| #31 | Intervention' OR 'non-pharmacological interventions' OR 'non-pharmaceutical interventions' |
| #32 | #23 OR #24 OR #25 OR #26 OR #27 OR #28 OR #29 OR #30 OR #31 |
| #33 | randomized:ti,ab,kw OR randomly:ti,ab,kw OR 'placebo':ti,ab,kw OR 'trial':ti,ab,kw OR groups:ti,ab,kw OR control:ti,ab,kw |
| #34 | #22 AND #32 AND #33 |
| #35 | 'nonhuman'/de OR 'human cell'/de |
| #36 | #34NOT #35 |
| #37 | #36 AND ('article'/it OR 'article in press'/it OR 'review'/it) |

**Medline**

| **No.** | **Query** |
| --- | --- |
| #1 | MHX=(Prehypertension) OR TS=(Prehypertension OR "High normal blood pressure" OR "High risk of hypertension" OR "Elevated blood pressure" OR "120-139 / 80-89 mmHg" OR "130-139 / 80-89 mmHg") |
| #2 | TS=("raised blood pressure") |
| #3 | MHX=("Masked hypertension" OR "white coat hypertension" OR "Pregnancy Induced Hypertension" OR "Pulmonary Hypertension" OR "Renal Hypertension" OR "Hypertensive Retinopathy" OR (animals NOT humans)) |
| #4 | TS=("Masked hypertension" OR "secondary hypertension" OR "white coat hypertension" OR pregnancy OR Pulmonary OR Renal OR Retinopathy OR rats OR mouse OR rabbits OR dogs ) |
| #5 | TI=("Influencing factors" OR "risk factors" OR Mechanism OR protocol) |
| #6 | TS=("intervention" OR "non-pharmacological interventions" OR "non-pharmaceutical interventions") |
| #7 | TS=(Diet OR "Dietary pattern" OR DASH OR salt OR sodium OR alcohol OR cigarette OR tobacco OR smoking OR nutrition OR potassium) |
| #8 | TS=("Sleep duration" OR "Sleep quality" OR "daytime sleepiness" OR "Afternoon napping") |
| #9 | TS=(Lifestyle OR exercise OR training OR "Weight loss" OR Yoga OR Qigong OR "Tai Ji" OR "tai chi" OR Meditation) |
| #10 | TS=("Health education" OR "Health Promotion" OR "Health Screening Program" OR "Health Services Accessibility" OR "Health Services Accessibility" OR "Patient Education" OR "Knowledge Attitude/Belief and Practice" OR KAP OR "Health Knowledge, Attitudes, Practice") |
| #11 | TS=(self-care OR self-management OR Acupuncture OR "Mental Health" OR "Self Report" OR "Self Efficacy") |
| #12 | TS=("wearable devices" OR smartphone OR wechat OR "smart devices" OR telehealth OR "Electronic health" OR "online health community" OR "ambulatory blood pressure monitoring" OR Telemedicine OR "online health") |
| #13 | TS=("family doctor" OR "multidisciplinary team" OR "coordinated service" OR "continuity of care" OR "Medical union" OR "medical alliance" OR "integrated care") |
| #14 | TS=(payment OR incentives OR reimbursement OR "delivery system") |
| #15 | DT=("Clinical Study" OR "Clinical Trial" OR "Comparative Study" OR "Controlled Clinical Trial" OR "Randomized Controlled Trial") |
| #16 | TS=(randomized OR randomly OR placebo OR trial OR groups OR cohort OR longitudinal OR prospective OR retrospective) |
| #17 | (#1) OR #2 |
| #18 | ((#3) OR #4) OR #5 |
| #19 | ((((((((#6) OR #7) OR #8) OR #9) OR #10) OR #11) OR #12) OR #13) OR #14 |
| #20 | (#15) OR #16 |
| #21 | (((#17) NOT #18) AND #19) AND #20 |
| #22 | ((((#1) OR #2) NOT (((#3) OR #4) OR #5)) AND (((((((((#6) OR #7) OR #8) OR #9) OR #10) OR #11) OR #12) OR #13) OR #14)) AND ((#15) OR #16) |
