# Supplementary material for: Short-term efficacy of non-pharmacological interventions for global population with elevated blood pressure: A network meta-analysis
Source: Front Public Health. 2023 Jan 13;10:1051581. doi: 10.3389/fpubh.2022.1051581 (PMC9880179; doi:10.3389/fpubh.2022.1051581)
Supplement: Supplementary material 2 — Main characteristics of included trials. [file Table_2.DOCX]

**Supplemental material 2: Main characteristics of included trials**

| Main characteristics of included trials | | | | | | | | | | | | |
| --- | --- | --- | --- | --- | --- | --- | --- | --- | --- | --- | --- | --- |
| First Author, Year | Average age | | Country | Sample Size | | Male(%) | | Intervention | | Baseline BP | | Follow up |
|  | I | C |  | I | C | I (%) | C (%) | I | C | I | C |  |
| Thiyagarajan 2015 | 44.08±9.42 | 42.47±9.00 | India | 51 | 49 | 60.78% | 63.27% | yoga | lifestyle | 127 / 85 | 127 / 85 | 12weeks |
| Ankolekar 2019 | / | / | India | 51 | 51 | / | / | yoga | usual care | 134 / 85.95 | 133.45 / 86.03 | 6months |
| Hughes 2013 | 51.2 ±5.8 | 49.5 ±7.2 | Canada | 24 | 32 | 45.83% | 40.63% | meditation | normal exercise | 130.2 / 77.3 | 128.8 / 78.3 | 12weeks |
| Staffileno 2018 | 35.3±8.1 | 35.1±1.6 | USA | 14 | 12 | 0.00% | 0.00% | DASH | Lifestyle | 122.9 / 83.6 | 123.3 / 82.8 | 12weeks |
| Juraschek 2018 | 59.2 ±8.4 | 58.8 ±10.5 | USA | 60 | 57 | 35.00% | 24.56% | DASH | usual care | 130 / 76.4 | 133.1 / 78 | 8weeks |
| Allaert 2017 | 51.0±16.0 | | / | 22 | 19 | / | / | salt restriction | usual care | 133.8 / 79.5 | 136.6 / 82.6 | 8weeks |
| Naseem 2016 | 54.41±9.367 | 52.32±9.110 | Pakistan | 710 | 782 | 55.21% | 40.66% | salt restriction | usual care | 128.52 / 84.52 | 128.21 / 84.2 | 6months |
| Ogbutor 2019 | 40.78±6.04 | 41.27±6.31 | USA | 200 | 200 | 54.50% | 56.00% | isometric exercise | Lifestyle | 133.52 / 87.68 | 126.24 / 81.63 | 24days |
| Goessler 2017 | 33.1±2.1 | 32.9±2.1 | / | 22 | 22 | 45.45% | 63.64% | isometric exercise | aerobic exercise | 125.2 / 84.6 | 125.8 / 85.4 | 8weeks |
| Katrina 2018 | 43.8±7.3 | | UK | 12 | 12 | / | / | isometric exercise | usual care | 132.4 / 81.4 | 132.2 / 81.7 | 12months |
| Jessup 1998 | 67.8±4.4 | 69.2±5.1 | USA | 11 | 10 | 45.45% | 50.00% | aerobic exercise | usual care | 132.6 / 79.8 | 130.8 / 80.2 | 16weeks |
| Kamalakkannan 2014 | 18-20 | | India | 20 | 20 | 100.00% | 100.00% | aerobic exercise | usual care | 122.1 / 82.9 | 123.3 / 82.1 | 12weeks |
| Kim 2012 | 54.53±2.82 | | South Korea | 15 | 15 | 0.00% | 0.00% | aerobic exercise | usual care | 133.23 / 86.08 | 131.62 / 86.92 | 16weeks |
| Tantiprasoplap 2018 | 55.9±7.3 | 54.4±6.7 | Thailand | 42 | 42 | 0.00% | 0.00% | aerobic exercise | usual care | 131.38 / 72.48 | 134.26 / 75.33 | 6months |
| Azadpour 2016 | 57.58±4.29 | 56.58±4.17 | USA | 12 | 12 | 0.00% | 0.00% | aerobic exercise | usual care | 127.92 / 82 | 129.58 / 82.33 | 10weeks |
| Glodzik 2018 | 44.3 ± 5.57 | 45.0 ± 3.41 | Poland | 31 | 14 | 74.19% | 57.14% | aerobic exercise | usual care | 131.65 / 80.65 | 131.66 / 83 | 12weeks |
| Son 2019 | 67.7±1.0 | 67.4±1.1 | South Korea | 10 | 10 | / | / | resistance exercise | usual care | 138.5 / 81.7 | 138.9 / 81.4 | 12weeks |
| Pengpid 2019 | 35-65 | 35-65 | Thailand | 220 | 223 | 25.45% | 26.01% | Lifestyle | usual care | 126.7 / 79.8 | 126.6 / 79 | 12months |
| Neupane 2018 | 46.02±9.73 | 45.15±9.92 | Nepal | 175 | 128 | 30.29% | 31.25% | Lifestyle | usual care | 124.27 / 81.76 | 125.5 / 82.44 | 12months |
| Zhao 2014 | 43.49±9.35 | 43.94±9.52 | China | 278 | 278 | 46.04% | 55.04% | Lifestyle | usual care | 124.78 / 81.77 | 124.31 / 81.81 | 12months |
| Celedonio 2009 | 43.97±7.65 | 42.56±7.98 | Mexico | 38 | 43 | / | / | Lifestyle | usual care | 133.03 / 87.58 | 132.72 / 85.6 | 6months |
| Seo 2021* | 57.44 ±3.71 | 57.87±4.42 | South Korea | 61 | 61 | 0.00% | 0.00% | acupuncture | usual care | 130.2 / 84.6 | 128.4 / 83.9 | 24months |
| Shin 2019 | 40.93±15.91 | 40.87±13.96 | South Korea | 15 | 15 | 60.00% | 73.33% | acupuncture | usual care | 129.59 / 79.7 | 132.39 / 82.96 | 4weeks |
| Lim 2015 | 56.80±1.82 | 58.33±1.88 | South Korea | 15 | 15 | 100.00% | 100.00% | normal exercise | usual care | 133.1 / 89.07 | 131.5 / 85.47 | 10weeks |
| Whelton 1995 | 42.8±6.5 | 43.1±6.6 | USA | 178 | 175 | 74.72% | 69.71% | high Potassium | usual care | 120.7 / 80.8 | 122.6 / 81.1 | 6months |
| Zilkens 2003 | 28-65 | | Australia | 16 | | 100.00% | | Reduced alcohol | usual care | 125 / 77 | 125 / 77 | 8weeks |
| Rakic 1998 | 48.2±1.3 | | Australia | 41 | | 100.00% | | Reduced alcohol | usual care | 124 / 77 | 124 / 77 | 12weeks |
| Parker 2015 | 45.4±2.35 | 44.8±2.17 | Australia | 20 | 21 | 100.00% | 100.00% | Reduced alcohol | usual care | 137.6 / 83.8 | 136 / 85.5 | 18weeks |
| Márquez 2018 | 55.7±10.2 | 57.1±5.4 | Spain | 24 | 18 | 45.80% | 38.90% | meditation | usual care | 136.41 / 87.88 | 131.34 / 85.09 | 8weeks |
| Wenneberg 1997 | 18-34 | | USA | 14 | 12 | 100.00% | 100.00% | meditation | usual care | 128.8 / 70.6 | 128.8 / 70.6 | 4months |
| Stevens 1993* | 43.1±6.0 | 42.4±6.2 | USA | 308 | 256 | 72.73% | 62.89% | weight loss | usual care | 124.3 / 83.7 | 124.6 / 84 | 6months |
| Rubinstein 2015* | 43.6±8.4 | 43.2±8.4 | Multi-center | 316 | 321 | 47.00% | 46.00% | Lifestyle | usual care | 127.2 / 77.4 | 127.3 / 77.5 | 6months |
| Anderssen 1995 | 44.6±2.89 | | Norway | 209 | | / | | DASH | usual care | 133.6 / 88.1 | 129.6 / 88 | 12months |
|  |  |  |  |  |  |  |  | aerobic exercise |  | 130.6 / 88.2 |  |  |
| HPTRG 1990* | 38.3 | 38.5 | Multi-center | 195 | 196 | 65.10% | 61.20% | high Potassium | usual care | 124.1 / 82.3 | 123.9 / 83 | 6months |
|  | 39 |  |  | 195 |  | 63.30% |  | salt restriction |  | 124 / 82.6 |  |  |
| Beck 2014 | 21.1±0.6 | 21.6±0.8 | USA | 15 | 15 | 73.33% | 66.67% | resistance exercise | usual care | 130 / 80 | 130 / 81 | 8weeks |
|  | 20.1±0.9 |  |  | 13 |  | 69.23% |  | combination exercise |  | 132 / 81 |  |  |
| TTHPCRG 1997* | 44.2±6.1 | 43.2±6.1 | Multi-center | 594 | 596 | 64.80% | 68.30% | salt restriction | usual care | 127.7 / 86.1 | 127.3 / 85.8 | 6months |
|  | 43.4±6.1 |  |  | 595 |  | 63.00% |  | weight loss |  | 127.61 / 86 |  |  |
| Puska 1983 | 30-50 | | Karelia | 34 | 38 | 50.00% | | salt restriction | usual care | 132 / 82.7 | 129.5 / 81.3 | 12weeks |
|  |  |  |  | 35 |  |  |  | weight loss |  | 127.2 / 80.1 |  |  |
| Saptharishi 2009 | 22.4±1.3 | 22.5±1.4 | India | 28 | 30 | 70.37% | 66.67% | normal exercise | usual care | 128.6 / 87.4 | 123.1 / 82.9 | 8weeks |
|  | 22.5±1.47 |  |  | 28 |  | 60.00% |  | salt restriction |  | 124 / 83.7 |  |  |
|  | 22.5±1.36 |  |  | 27 |  | 66.67% |  | yoga |  | 126.8 / 84.5 |  |  |
| Baross 2017 | 20.7±1.6 | 21.3±2.0 | UK | 12 | 12 | 50.00% | 58.33% | combination exercise | usual care | 127.8 / 77.2 | 127.9 / 77 | 6weeks |
|  | 20.9±2.0 |  |  | 12 |  | 50.00% |  | isometric exercise |  | 127.1 / 76.3 |  |  |
|  | 20.0±0.5 |  |  | 12 |  | 58.33% |  | normal exercise |  | 126.7 / 77.7 |  |  |
| Notes :Studies with * contain follow-up longer than 12 months, but we would extract short-term BP changes if they included follow-up reports within a year. | | | | | | | | | | | | |
