# Supplementary material for: Short-term efficacy of non-pharmacological interventions for global population with elevated blood pressure: A network meta-analysis
Source: Front Public Health. 2023 Jan 13;10:1051581. doi: 10.3389/fpubh.2022.1051581 (PMC9880179; doi:10.3389/fpubh.2022.1051581)
Supplement: Supplementary material 3 — Results of node-splitting and heterogeneity test. [file Table_3.DOCX]

**Supplemental material 3: Node-splitting and Heterogeneity test**

Node-splitting results of SBP in intervention items


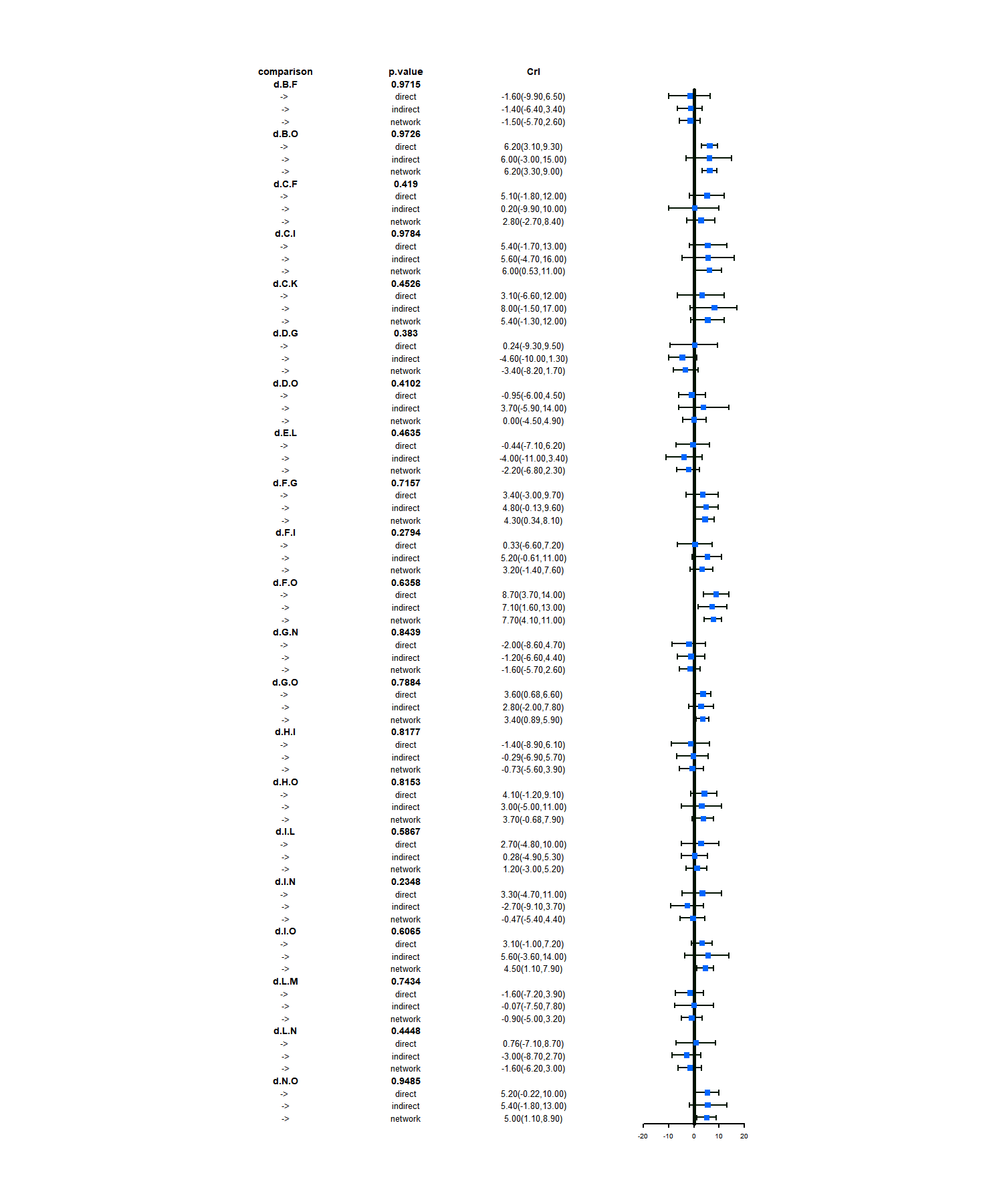


Node-splitting results of DBP in intervention items


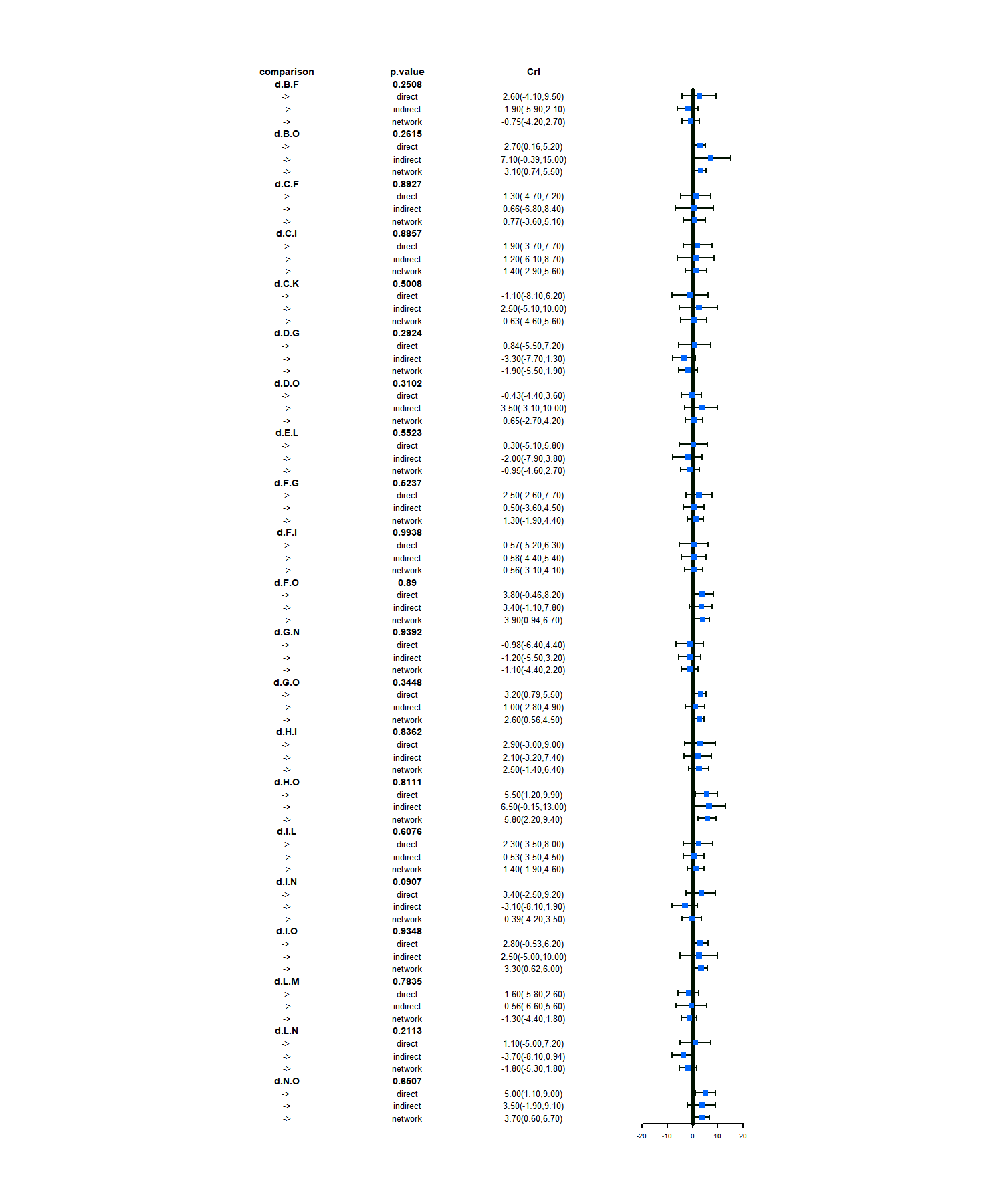


Node-splitting results of SBP in intervention groups


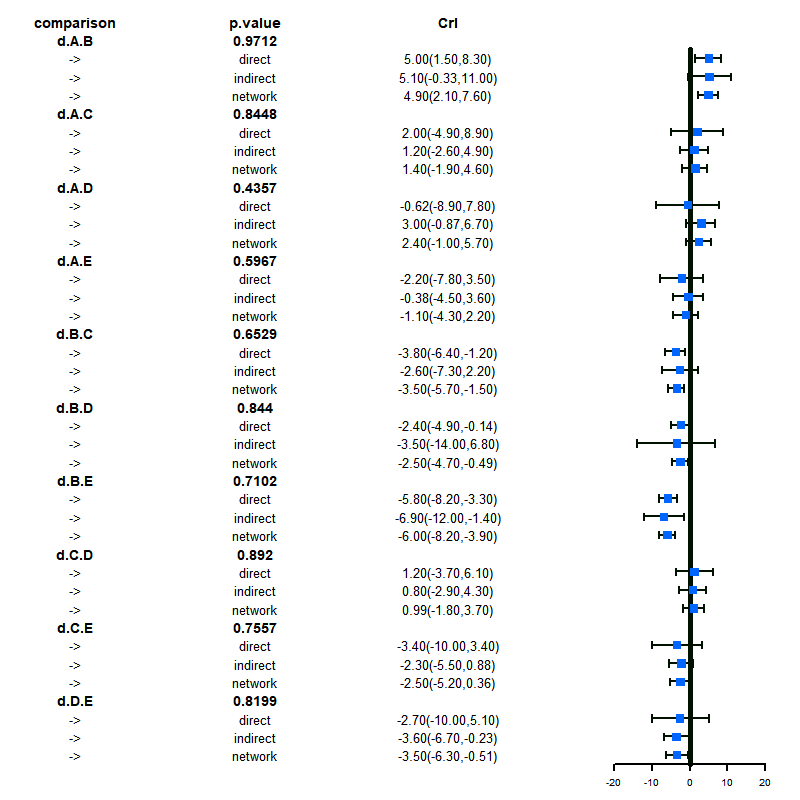


Node-splitting results of DBP in intervention groups


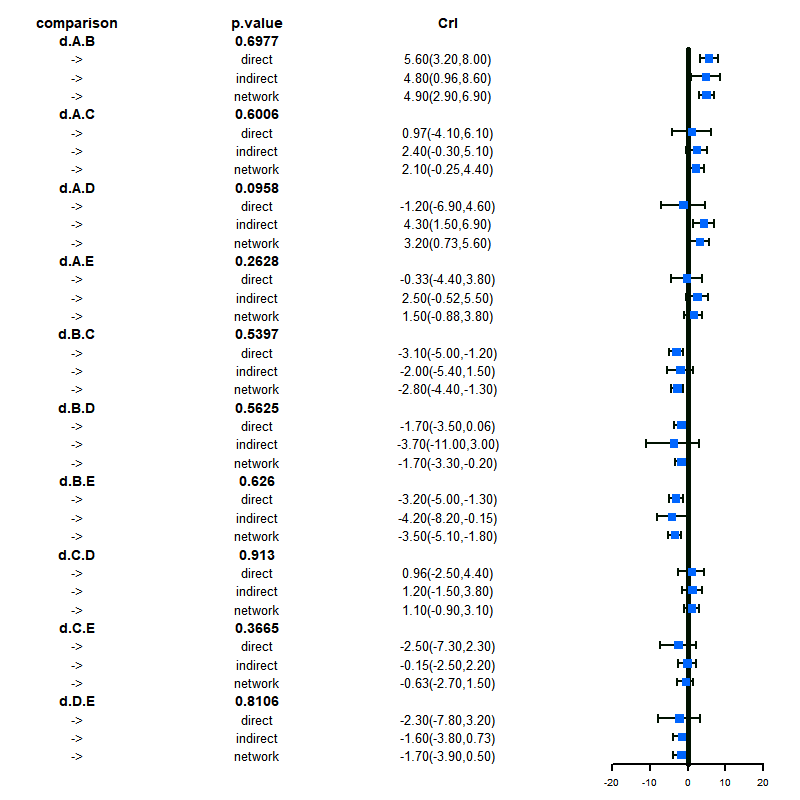


Heterogeneity test of intervention items

| Treatment | | SBP | | | DBP | | |
| --- | --- | --- | --- | --- | --- | --- | --- |
| 1 | 2 | i2.pair | i2.cons | incons.p | i2.pair | i2.cons | incons.p |
| A | O | 0.00 | 0.00 | NA | 3.00 | 5.87 | NA |
| B | F | NA | 0.00 | 0.97 | NA | 57.54 | 0.26 |
| B | O | 67.86 | 61.47 | 0.98 | 79.79 | 77.64 | 0.30 |
| C | F | NA | 25.07 | 0.41 | NA | 0.00 | 0.84 |
| C | I | NA | 0.00 | 0.84 | NA | 0.00 | 0.82 |
| C | K | NA | 0.00 | 0.53 | NA | 0.00 | 0.60 |
| C | O | 0.00 | 0.00 | NA | 20.36 | 24.27 | NA |
| D | G | NA | 8.57 | 0.40 | NA | 53.53 | 0.31 |
| D | O | 96.39 | 95.27 | 0.50 | 92.42 | 94.56 | 0.35 |
| E | L | NA | 68.43 | 0.47 | NA | 55.33 | 0.54 |
| E | O | 39.28 | 74.34 | NA | 0.00 | 40.24 | NA |
| F | G | NA | 71.02 | 0.74 | NA | 96.72 | 0.55 |
| F | I | NA | 50.83 | 0.37 | NA | 0.00 | 0.98 |
| F | O | 88.01 | 83.04 | 0.45 | 62.58 | 25.38 | 0.93 |
| G | N | NA | 0.00 | 0.87 | NA | 0.00 | 0.95 |
| G | O | 89.64 | 86.42 | 0.83 | 94.66 | 93.33 | 0.40 |
| H | I | NA | 0.00 | 0.82 | NA | 0.00 | 0.85 |
| H | O | 95.27 | 91.26 | 0.88 | 0.00 | 0.00 | 0.79 |
| I | L | NA | 0.00 | 0.67 | NA | 0.00 | 0.74 |
| I | N | NA | 28.03 | 0.35 | NA | 76.41 | 0.16 |
| I | O | 62.51 | 77.08 | 0.23 | 51.77 | 45.48 | 0.59 |
| J | O | 56.52 | 56.34 | NA | 0.00 | 0.00 | NA |
| K | O | 16.00 | 39.31 | NA | 68.59 | 72.43 | NA |
| L | M | 0.00 | 0.00 | 0.80 | 0.00 | 0.00 | 0.86 |
| L | N | NA | 0.00 | 0.63 | NA | 23.55 | 0.37 |
| L | O | 90.25 | 87.15 | NA | 96.71 | 96.59 | NA |
| M | O | 0.00 | 0.00 | NA | 0.00 | 13.28 | NA |
| N | O | 48.32 | 53.83 | 0.65 | 69.40 | 86.31 | 0.25 |
| Notes: (1) Cons: Consistency effect; (2) Pair: Pair-wise pooled effect; (3) incons.p: P value of inconsistency | | | | | | | |

Heterogeneity test of intervention items

| Treatment | | SBP | | | DBP | | |
| --- | --- | --- | --- | --- | --- | --- | --- |
| 1 | 2 | i2.pair | i2.cons | incons.p | i2.pair | i2.cons | incons.p |
| A | B | 73.34 | 70.89 | 0.84 | 0.00 | 43.79 | 0.25 |
| A | C | NA | 0.00 | 0.87 | NA | 55.03 | 0.61 |
| A | D | NA | 0.00 | 0.55 | NA | 62.00 | 0.20 |
| A | E | 0.00 | 0.00 | 0.66 | 83.81 | 71.74 | 0.40 |
| B | C | 82.77 | 79.18 | 0.76 | 90.77 | 89.22 | 0.63 |
| B | D | 92.15 | 91.67 | 0.84 | 94.16 | 94.64 | 0.52 |
| B | E | 84.37 | 82.82 | 0.69 | 71.98 | 71.71 | 0.38 |
| C | D | 0.00 | 0.00 | 0.99 | 0.00 | 0.00 | 0.83 |
| C | E | NA | 72.96 | 0.77 | NA | 98.68 | 0.37 |
| D | E | NA | 0.00 | 0.85 | NA | 0.00 | 0.84 |
| Notes: (1) Cons: Consistency effect; (2) Pair: Pair-wise pooled effect; (3) incons.p: P value of inconsistency | | | | | | | |
