# Supplementary material for: Short-term efficacy of non-pharmacological interventions for global population with elevated blood pressure: A network meta-analysis
Source: Front Public Health. 2023 Jan 13;10:1051581. doi: 10.3389/fpubh.2022.1051581 (PMC9880179; doi:10.3389/fpubh.2022.1051581)

**Supplemental material 4: Risk of bias assessment**

**Risk of bias assessment diagram:**


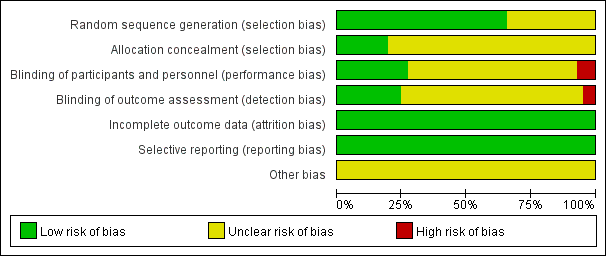


**Risk of bias summary:**


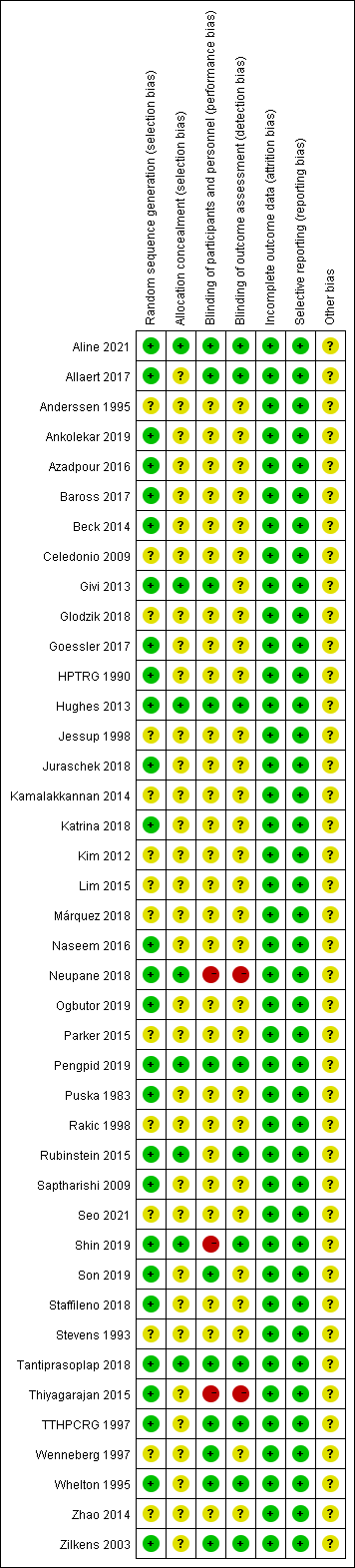

Supplement: Supplementary material 4 — “Quality assessment diagram” and “Risk of bias summary.” [file Table_4.DOCX]
