# Supplementary material for: Short-term efficacy of non-pharmacological interventions for global population with elevated blood pressure: A network meta-analysis
Source: Front Public Health. 2023 Jan 13;10:1051581. doi: 10.3389/fpubh.2022.1051581 (PMC9880179; doi:10.3389/fpubh.2022.1051581)
Supplement: Supplementary material 5 — Detailed results of the certainty of the evidence analysis. [file Table_5.DOCX]

**Supplemental material 5: Detailed results of the certainty of the evidence analysis**

**A summary description of the certainty of the evidence analysis**

A recommended 4-step approach was used in this study. In the first step, the effect sizes and confidence intervals of the direct evidence, indirect evidence and network meta-analysis evidence were presented separately. In the second step, the quality of the direct evidence for each comparison group was graded without considering the imprecision. If the direct evidence was graded "high" and the contribution to the network meta-analysis results was greater than or equal to indirect evidence, no indirect evidence quality grading was required. The network meta-analysis evidence quality was directly assessed based on the direct evidence quality. Otherwise, indirect evidence quality grading was required. In the third step, based on the quality of direct evidence in the first-order loop of indirect evidence, the quality of indirect evidence was determined. The intransitivity should also be considered. In the fourth step, based on the level of direct evidence and/or indirect evidence, and considering inconsistency and imprecision, the quality of evidence for network meta-analysis was finalized and presented.

**Serious risk of bias:** Total three studies existed serious risk of bias (see **Supplemental material 5** for more information). Two studies were small sample size studies (smaller than 100), the influence can be ignored. The other big sample size study (Neupane 2018, more than 300) had influence on analysis of interventions, but with the influence on intervention groups being ignored.

**Serious Indirectness:** The standardization of interventions in this study were carried out following the guidelines, still it may leave to subjectivity. This meant that the included interventions may have not sufficiently difference with targeted interventions. The differences in population baseline of included studies could not be ignored either. Therefore, most comparisons were downgraded in the indirectness of evidence due to the differences in population and intervention.

**Intransitivity:** When dealing with indirect evidence quality grading, it is necessary to pay attention to whether there are significant differences between different groups in terms of population baseline characteristics, common controls, and outcome measures.

**Imprecision:** Judgments of imprecision were made using a minimally contextualised approach, with a null effect as the threshold of importance. Imprecision was judged after the judgement of evidence contribution.

**Heterogeneity:** Judged according to the pairwise meta-analysis from the network meta-analysis.

**Direct evidence and indirect evidence contribution:** The GRADE working group recommends that the contribution of direct evidence and indirect evidence to NMA results be judged by the width of the confidence interval, and evidence with a narrower confidence interval contributes more to the NMA.

**Incoherence:** Downgrade if there is significant inconsistency between direct and indirect comparative evidence.

**Table 1 Certainty of the evidence in the SBP reduction of Interventions**

| **Name** | **ID** | **Direct evidence**  **MD (95%CI)** | **Evidence level** | **Indirect evidence**  **MD (95%CI)** | **Evidence level** | **Network evidence**  **MD (95%CI)** | **Evidence level** |
| --- | --- | --- | --- | --- | --- | --- | --- |
| acupuncture vs usual care | A vs O | -6.95 (-11.26, -2.64)* | ⨁⨁⨁◯Moderate | ↑↑ | - | -6.57 (-13.21, 0.15)↥ | ⨁⨁⨁◯Moderate |
| aerobic exercise vs usual care | B vs O | -6.2 (-9.3, -3.1)*§ | ⨁⨁◯◯Low | -6 (-15, 3)* | ⨁⨁⨁◯Moderate | -6.18 (-9.06, -3.29)↥ | ⨁⨁◯◯Low |
| combination exercise vs usual care | C vs O | -10.17 (-13.32, -7.03)* | ⨁⨁⨁◯Moderate | -10.51 (-15.44, -5.54)* | ⨁⨁⨁◯Moderate | -10.51 (-15.44, -5.54)↥ | ⨁⨁⨁◯Moderate |
| DASH vs usual care | D vs O | 0.95 (-4.5, 6)⇟§ | ⨁⨁◯◯Low | -3.7 (-14, 5.9)* | ⨁⨁⨁◯Moderate | -0.02 (-4.86, 4.4)↥ | ⨁⨁◯◯Low |
| high Potassium vs usual care | E vs O | -0.40 (-1.41, 0.61)*⇟ | ⨁⨁⨁◯Moderate | -1.14 (-5.32, 3.05)* | ⨁⨁⨁◯Moderate | -1.14 (-5.32, 3.05)↥ | ⨁⨁◯◯Low |
| isometric exercise vs usual care | F vs O | -8.7 (-14, -3.7)*§ | ⨁⨁◯◯Low | -7.1 (-13, -1.6)* | ⨁⨁⨁◯Moderate | -7.65 (-11.13, -4.25)↥ | ⨁⨁◯◯Low |
| Lifestyle vs usual care(bias) | G vs O | -3.6 (-6.6, -0.68)†§ | ⨁⨁◯◯Low | -2.8 (-7.8, 2)†* | ⨁⨁◯◯Low | -3.4 (-5.89, -0.94)↥ | ⨁⨁◯◯Low |
| meditation vs usual care | H vs O | -4.1 (-9.1, 1.2)*⇟§ | ⨁⨁◯◯Low | -3 (-11, 5)* | ⨁⨁⨁◯Moderate | -3.81 (-7.98, 0.7)↥ | ⨁◯◯◯Very low |
| normal exercise vs usual care | I vs O | -3.1 (-7.2, 1)*⇟§ | ⨁⨁◯◯Low | -5.6 (-14, 3.6)* | ⨁⨁⨁◯Moderate | -4.53 (-7.89, -1.15)↥ | ⨁◯◯◯Very low |
| reduced alcohol vs usual care | J vs O | -2.28 (-3.17, -1.40)*§ | ⨁⨁◯◯Low | ↑↑ | - | -3.03 (-7.22, 1.14)↥ | ⨁⨁◯◯Low |
| resistance exercise vs usual care | K vs O | -3.93 (-7.37, -0.48)* | ⨁⨁⨁◯Moderate | -5.05 (-10.71, 0.52)* | ⨁⨁⨁◯Moderate | -5.05 (-10.71, 0.52)↥ | ⨁⨁⨁◯Moderate |
| salt restriction vs usual care | L vs O | -2.46 (-2.80, -2.12)*§ | ⨁⨁◯◯Low | -3.3 (-6.02, -0.7)* | ⨁⨁⨁◯Moderate | -3.3 (-6.02, -0.7)↥ | ⨁⨁◯◯Low |
| weight loss vs usual care | M vs O | -3.82 (-4.60, -3.03)* | ⨁⨁⨁◯Moderate | -4.23 (-7.97, -0.58)* | ⨁⨁⨁◯Moderate | -4.23 (-7.97, -0.58)↥ | ⨁⨁⨁◯Moderate |
| yoga vs usual care | N vs O | -5.2 (-10, 0.22)*⇟ | ⨁⨁⨁◯Moderate | -5.4 (-13, 1.8)* | ⨁⨁⨁◯Moderate | -4.98 (-8.8, -0.98)↥ | ⨁⨁◯◯Low |
| acupuncture vs yoga | A vs N | - | - | -1.59 (-9.3, 6.1)*⇟ | ⨁⨁⨁◯Moderate | -1.59 (-9.3, 6.1)↧ | ⨁⨁◯◯Low |
| aerobic exercise vs yoga | B vs N | - | - | -1.2 (-6.11, 3.57)*⇟ | ⨁⨁⨁◯Moderate | -1.2 (-6.11, 3.57)↧ | ⨁⨁◯◯Low |
| combination exercise vs yoga | C vs N | - | - | -5.54 (-11.8, 0.71)*⇟ | ⨁⨁⨁◯Moderate | -5.54 (-11.8, 0.71)↧ | ⨁⨁◯◯Low |
| DASH vs yoga | D vs N | - | - | 4.96 (-1.3, 10.68)*⇟ | ⨁⨁⨁◯Moderate | 4.96 (-1.3, 10.68)↧ | ⨁⨁◯◯Low |
| high Potassium vs yoga | E vs N | - | - | 3.82 (-1.92, 9.49)*⇟ | ⨁⨁⨁◯Moderate | 3.82 (-1.92, 9.49)↧ | ⨁⨁◯◯Low |
| isometric exercise vs yoga | F vs N | - | - | -2.67 (-7.8, 2.3)*⇟ | ⨁⨁⨁◯Moderate | -2.67 (-7.8, 2.3)↧ | ⨁⨁◯◯Low |
| Lifestyle vs yoga | G vs N | 2 (-4.7, 8.6)* | ⨁⨁⨁◯Moderate | 1.2 (-4.4, 6.6)*⇟ | ⨁⨁⨁◯Moderate | 1.59 (-2.65, 5.64)↧ | ⨁⨁◯◯Low |
| meditation vs yoga | H vs N | - | - | 1.18 (-4.5, 6.99)*⇟ | ⨁⨁⨁◯Moderate | 1.18 (-4.5, 6.99)↧ | ⨁⨁◯◯Low |
| normal exercise vs yoga | I vs N | -3.3 (-11, 4.7)* | ⨁⨁⨁◯Moderate | 2.7 (-3.7, 9.1)*⇟ | ⨁⨁⨁◯Moderate | 0.47 (-4.45, 5.22)↧ | ⨁⨁◯◯Low |
| reduced alcohol vs yoga | J vs N | - | - | 1.98 (-3.88, 7.58)*⇟ | ⨁⨁⨁◯Moderate | 1.98 (-3.88, 7.58)↧ | ⨁⨁◯◯Low |
| resistance exercise vs yoga | K vs N | - | - | -0.07 (-7.04, 6.6)*⇟ | ⨁⨁⨁◯Moderate | -0.07 (-7.04, 6.6)↧ | ⨁⨁◯◯Low |
| salt restriction vs yoga | L vs N | -0.76 (-8.7, 7.1)* | ⨁⨁⨁◯Moderate | 3 (-2.7, 8.7)*⇟ | ⨁⨁⨁◯Moderate | 1.68 (-2.98, 6.12)↧ | ⨁⨁◯◯Low |
| weight loss vs yoga | M vs N | - | - | 0.76 (-4.66, 5.93)*⇟ | ⨁⨁⨁◯Moderate | 0.76 (-4.66, 5.93)↧ | ⨁⨁◯◯Low |
| acupuncture vs weight loss | A vs M | - | - | -2.34 (-9.89, 5.31)*⇟ | ⨁⨁⨁◯Moderate | -2.34 (-9.89, 5.31)↧ | ⨁⨁◯◯Low |
| aerobic exercise vs weight loss | B vs M | - | - | -1.94 (-6.57, 2.74)*⇟ | ⨁⨁⨁◯Moderate | -1.94 (-6.57, 2.74)↧ | ⨁⨁◯◯Low |
| combination exercise vs weight loss | C vs M | - | - | -6.28 (-12.35, -0.1)* | ⨁⨁⨁◯Moderate | -6.28 (-12.35, -0.1)↧ | ⨁⨁⨁◯Moderate |
| DASH vs weight loss | D vs M | - | - | 4.21 (-1.86, 9.9)*⇟ | ⨁⨁⨁◯Moderate | 4.21 (-1.86, 9.9)↧ | ⨁⨁◯◯Low |
| high Potassium vs weight loss | E vs M | - | - | 3.09 (-2.31, 8.61)*⇟ | ⨁⨁⨁◯Moderate | 3.09 (-2.31, 8.61)↧ | ⨁⨁◯◯Low |
| isometric exercise vs weight loss | F vs M | - | - | -3.43 (-8.44, 1.64)*⇟ | ⨁⨁⨁◯Moderate | -3.43 (-8.44, 1.64)↧ | ⨁⨁◯◯Low |
| Lifestyle vs weight loss | G vs M | - | - | 0.83 (-3.56, 5.25)*⇟ | ⨁⨁⨁◯Moderate | 0.83 (-3.56, 5.25)↧ | ⨁⨁◯◯Low |
| meditation vs weight loss | H vs M | - | - | 0.42 (-5.09, 6.31)*⇟ | ⨁⨁⨁◯Moderate | 0.42 (-5.09, 6.31)↧ | ⨁⨁◯◯Low |
| normal exercise vs weight loss | I vs M | - | - | -0.31 (-5.17, 4.67)*⇟ | ⨁⨁⨁◯Moderate | -0.31 (-5.17, 4.67)↧ | ⨁⨁◯◯Low |
| reduced alcohol vs weight loss | J vs M | - | - | 1.21 (-4.35, 6.8)*⇟ | ⨁⨁⨁◯Moderate | 1.21 (-4.35, 6.8)↧ | ⨁⨁◯◯Low |
| resistance exercise vs weight loss | K vs M | - | - | -0.82 (-7.54, 5.84)*⇟ | ⨁⨁⨁◯Moderate | -0.82 (-7.54, 5.84)↧ | ⨁⨁◯◯Low |
| salt restriction vs weight loss | L vs M | 1.6 (-3.9, 7.2)*⇟ | ⨁⨁⨁◯Moderate | 0.067 (-7.8, 7.5)* | ⨁⨁⨁◯Moderate | 0.91 (-3.1, 4.94)↥ | ⨁⨁◯◯Low |
| acupuncture vs salt restriction | A vs L | - | - | -3.26 (-10.38, 3.98)*⇟ | ⨁⨁⨁◯Moderate | -3.26 (-10.38, 3.98)↧ | ⨁⨁◯◯Low |
| aerobic exercise vs salt restriction | B vs L | - | - | -2.86 (-6.8, 1.09)*⇟ | ⨁⨁⨁◯Moderate | -2.86 (-6.8, 1.09)↧ | ⨁⨁◯◯Low |
| combination exercise vs salt restriction | C vs L | - | - | -7.2 (-12.75, -1.57)* | ⨁⨁⨁◯Moderate | -7.2 (-12.75, -1.57)↧ | ⨁⨁⨁◯Moderate |
| DASH vs salt restriction | D vs L | - | - | 3.27 (-2.19, 8.45)*⇟ | ⨁⨁⨁◯Moderate | 3.27 (-2.19, 8.45)↧ | ⨁⨁◯◯Low |
| high Potassium vs salt restriction | E vs L | 0.44 (-6.2, 7.1)*⇟ | ⨁⨁⨁◯Moderate | 4 (-3.4, 11)* | ⨁⨁⨁◯Moderate | 2.17 (-2.32, 6.75)↥ | ⨁⨁◯◯Low |
| isometric exercise vs salt restriction | F vs L | - | - | -4.35 (-8.64, 0.02)*⇟ | ⨁⨁⨁◯Moderate | -4.35 (-8.64, 0.02)↧ | ⨁⨁◯◯Low |
| Lifestyle vs salt restriction | G vs L | - | - | -0.09 (-3.68, 3.5)*⇟ | ⨁⨁⨁◯Moderate | -0.09 (-3.68, 3.5)↧ | ⨁⨁◯◯Low |
| meditation vs salt restriction | H vs L | - | - | -0.5 (-5.38, 4.75)*⇟ | ⨁⨁⨁◯Moderate | -0.5 (-5.38, 4.75)↧ | ⨁⨁◯◯Low |
| normal exercise vs salt restriction | I vs L | -2.7 (-10, 4.8)* | ⨁⨁⨁◯Moderate | -0.28 (-5.3, 4.9)*⇟ | ⨁⨁⨁◯Moderate | -1.21 (-5.26, 2.87)↧ | ⨁⨁◯◯Low |
| reduced alcohol vs salt restriction | J vs L | - | - | 0.3 (-4.71, 5.28)*⇟ | ⨁⨁⨁◯Moderate | 0.3 (-4.71, 5.28)↧ | ⨁⨁◯◯Low |
| resistance exercise vs salt restriction | K vs L | - | - | -1.73 (-7.94, 4.4)*⇟ | ⨁⨁⨁◯Moderate | -1.73 (-7.94, 4.4)↧ | ⨁⨁◯◯Low |
| acupuncture vs resistance exercise | A vs K | - | - | -1.51 (-10.15, 7.24)*⇟ | ⨁⨁⨁◯Moderate | -1.51 (-10.15, 7.24)↧ | ⨁⨁◯◯Low |
| aerobic exercise vs resistance exercise | B vs K | - | - | -1.11 (-7.37, 5.25)*⇟ | ⨁⨁⨁◯Moderate | -1.11 (-7.37, 5.25)↧ | ⨁⨁◯◯Low |
| combination exercise vs resistance exercise | C vs K | -3.1 (-12, 6.6)* | ⨁⨁⨁◯Moderate | -8 (-17, 1.5)*⇟ | ⨁⨁⨁◯Moderate | -5.43 (-12, 1.21)↧ | ⨁⨁◯◯Low |
| DASH vs resistance exercise | D vs K | - | - | 5.02 (-2.3, 12.15)*⇟ | ⨁⨁⨁◯Moderate | 5.02 (-2.3, 12.15)↧ | ⨁⨁◯◯Low |
| high Potassium vs resistance exercise | E vs K | - | - | 3.93 (-2.99, 10.94)*⇟ | ⨁⨁⨁◯Moderate | 3.93 (-2.99, 10.94)↧ | ⨁⨁◯◯Low |
| isometric exercise vs resistance exercise | F vs K | - | - | -2.59 (-9.05, 3.9)*⇟ | ⨁⨁⨁◯Moderate | -2.59 (-9.05, 3.9)↧ | ⨁⨁◯◯Low |
| Lifestyle vs resistance exercise | G vs K | - | - | 1.64 (-4.39, 7.84)*⇟ | ⨁⨁⨁◯Moderate | 1.64 (-4.39, 7.84)↧ | ⨁⨁◯◯Low |
| meditation vs resistance exercise | H vs K | - | - | 1.25 (-5.62, 8.49)*⇟ | ⨁⨁⨁◯Moderate | 1.25 (-5.62, 8.49)↧ | ⨁⨁◯◯Low |
| normal exercise vs resistance exercise | I vs K | - | - | 0.53 (-5.9, 6.98)*⇟ | ⨁⨁⨁◯Moderate | 0.53 (-5.9, 6.98)↧ | ⨁⨁◯◯Low |
| reduced alcohol vs resistance exercise | J vs K | - | - | 2.03 (-4.92, 9.09)*⇟ | ⨁⨁⨁◯Moderate | 2.03 (-4.92, 9.09)↧ | ⨁⨁◯◯Low |
| acupuncture vs reduced alcohol | A vs J | - | - | -3.56 (-11.34, 4.35)*⇟ | ⨁⨁⨁◯Moderate | -3.56 (-11.34, 4.35)↧ | ⨁⨁◯◯Low |
| aerobic exercise vs reduced alcohol | B vs J | - | - | -3.17 (-8.2, 1.97)*⇟ | ⨁⨁⨁◯Moderate | -3.17 (-8.2, 1.97)↧ | ⨁⨁◯◯Low |
| combination exercise vs reduced alcohol | C vs J | - | - | -7.49 (-13.94, -0.94)* | ⨁⨁⨁◯Moderate | -7.49 (-13.94, -0.94)↧ | ⨁⨁⨁◯Moderate |
| DASH vs reduced alcohol | D vs J | - | - | 2.99 (-3.39, 9.03)*⇟ | ⨁⨁⨁◯Moderate | 2.99 (-3.39, 9.03)↧ | ⨁⨁◯◯Low |
| high Potassium vs reduced alcohol | E vs J | - | - | 1.9 (-3.99, 7.85)*⇟ | ⨁⨁⨁◯Moderate | 1.9 (-3.99, 7.85)↧ | ⨁⨁◯◯Low |
| isometric exercise vs reduced alcohol | F vs J | - | - | -4.64 (-10.04, 0.77)*⇟ | ⨁⨁⨁◯Moderate | -4.64 (-10.04, 0.77)↧ | ⨁⨁◯◯Low |
| Lifestyle vs reduced alcohol | G vs J | - | - | -0.39 (-5.21, 4.47)*⇟ | ⨁⨁⨁◯Moderate | -0.39 (-5.21, 4.47)↧ | ⨁⨁◯◯Low |
| meditation vs reduced alcohol | H vs J | - | - | -0.79 (-6.64, 5.42)*⇟ | ⨁⨁⨁◯Moderate | -0.79 (-6.64, 5.42)↧ | ⨁⨁◯◯Low |
| normal exercise vs reduced alcohol | I vs J | - | - | -1.52 (-6.87, 3.89)*⇟ | ⨁⨁⨁◯Moderate | -1.52 (-6.87, 3.89)↧ | ⨁⨁◯◯Low |
| acupuncture vs normal exercise | A vs I | - | - | -2.04 (-9.48, 5.42)*⇟ | ⨁⨁⨁◯Moderate | -2.04 (-9.48, 5.42)↧ | ⨁⨁◯◯Low |
| aerobic exercise vs normal exercise | B vs I | - | - | -1.64 (-6.02, 2.73)*⇟ | ⨁⨁⨁◯Moderate | -1.64 (-6.02, 2.73)↧ | ⨁⨁◯◯Low |
| combination exercise vs normal exercise | C vs I | -5.4 (-13, 1.7)*⇟ | ⨁⨁⨁◯Moderate | -5.6 (-16, 4.7)* | ⨁⨁⨁◯Moderate | -5.97 (-11.42, -0.55)↥ | ⨁⨁◯◯Low |
| DASH vs normal exercise | D vs I | - | - | 4.5 (-1.38, 10.04)*⇟ | ⨁⨁⨁◯Moderate | 4.5 (-1.38, 10.04)↧ | ⨁⨁◯◯Low |
| high Potassium vs normal exercise | E vs I | - | - | 3.4 (-1.9, 8.68)*⇟ | ⨁⨁⨁◯Moderate | 3.4 (-1.9, 8.68)↧ | ⨁⨁◯◯Low |
| isometric exercise vs normal exercise | F vs I | -0.33 (-7.2, 6.6)* | ⨁⨁⨁◯Moderate | -5.2 (-11, 0.61)*⇟ | ⨁⨁⨁◯Moderate | -3.12 (-7.56, 1.3)↧ | ⨁⨁◯◯Low |
| Lifestyle vs normal exercise | G vs I | - | - | 1.13 (-2.97, 5.14)*⇟ | ⨁⨁⨁◯Moderate | 1.13 (-2.97, 5.14)↧ | ⨁⨁◯◯Low |
| meditation vs normal exercise | H vs I | 1.4 (-6.1, 8.9)* | ⨁⨁⨁◯Moderate | 0.29 (-5.7, 6.9)*⇟ | ⨁⨁⨁◯Moderate | 0.73 (-3.89, 5.65)↧ | ⨁⨁◯◯Low |
| acupuncture vs meditation | A vs H | - | - | -2.8 (-10.84, 5.06)*⇟ | ⨁⨁⨁◯Moderate | -2.8 (-10.84, 5.06)↧ | ⨁⨁◯◯Low |
| aerobic exercise vs meditation | B vs H | - | - | -2.38 (-7.72, 2.68)*⇟ | ⨁⨁⨁◯Moderate | -2.38 (-7.72, 2.68)↧ | ⨁⨁◯◯Low |
| combination exercise vs meditation | C vs H | - | - | -6.7 (-13.26, -0.39)* | ⨁⨁⨁◯Moderate | -6.7 (-13.26, -0.39)↧ | ⨁⨁⨁◯Moderate |
| DASH vs meditation | D vs H | - | - | 3.78 (-2.97, 9.86)*⇟ | ⨁⨁⨁◯Moderate | 3.78 (-2.97, 9.86)↧ | ⨁⨁◯◯Low |
| high Potassium vs meditation | E vs H | - | - | 2.69 (-3.52, 8.5)*⇟ | ⨁⨁⨁◯Moderate | 2.69 (-3.52, 8.5)↧ | ⨁⨁◯◯Low |
| isometric exercise vs meditation | F vs H | - | - | -3.85 (-9.49, 1.42)*⇟ | ⨁⨁⨁◯Moderate | -3.85 (-9.49, 1.42)↧ | ⨁⨁◯◯Low |
| Lifestyle vs meditation | G vs H | - | - | 0.39 (-4.73, 5.19)*⇟ | ⨁⨁⨁◯Moderate | 0.39 (-4.73, 5.19)↧ | ⨁⨁◯◯Low |
| acupuncture vs Lifestyle | A vs G | - | - | -3.17 (-10.23, 4)*⇟ | ⨁⨁⨁◯Moderate | -3.17 (-10.23, 4)↧ | ⨁⨁◯◯Low |
| aerobic exercise vs Lifestyle | B vs G | - | - | -2.77 (-6.51, 1.01)*⇟ | ⨁⨁⨁◯Moderate | -2.77 (-6.51, 1.01)↧ | ⨁⨁◯◯Low |
| combination exercise vs Lifestyle | C vs G | - | - | -7.09 (-12.51, -1.61)* | ⨁⨁⨁◯Moderate | -7.09 (-12.51, -1.61)↧ | ⨁⨁⨁◯Moderate |
| DASH vs Lifestyle | D vs G | -0.24 (-9.5, 9.3)* | ⨁⨁⨁◯Moderate | 4.6 (-1.3, 10)*⇟ | ⨁⨁⨁◯Moderate | 3.37 (-1.75, 8.17)↧ | ⨁⨁◯◯Low |
| high Potassium vs Lifestyle | E vs G | - | - | 2.25 (-2.59, 7.16)*⇟ | ⨁⨁⨁◯Moderate | 2.25 (-2.59, 7.16)↧ | ⨁⨁◯◯Low |
| isometric exercise vs Lifestyle | F vs G | -3.4 (-9.7, 3)* | ⨁⨁⨁◯Moderate | -4.8 (-9.6, 0.13)*⇟ | ⨁⨁⨁◯Moderate | -4.24 (-8.03, -0.48)↧ | ⨁⨁◯◯Low |
| acupuncture vs isometric exercise | A vs F | - | - | 1.06 (-6.4, 8.71)*⇟ | ⨁⨁⨁◯Moderate | 1.06 (-6.4, 8.71)↧ | ⨁⨁◯◯Low |
| aerobic exercise vs isometric exercise | B vs F | 1.6 (-6.5, 9.9)* | ⨁⨁⨁◯Moderate | 1.4 (-3.4, 6.4)*⇟ | ⨁⨁⨁◯Moderate | 1.48 (-2.64, 5.69)↧ | ⨁⨁◯◯Low |
| combination exercise vs isometric exercise | C vs F | -5.1 (-12, 1.8)*⇟ | ⨁⨁⨁◯Moderate | -0.2 (-10, 9.9)* | ⨁⨁⨁◯Moderate | -2.85 (-8.31, 2.63)↥ | ⨁⨁◯◯Low |
| DASH vs isometric exercise | D vs F | - | - | 7.63 (1.7, 13.1)* | ⨁⨁⨁◯Moderate | 7.63 (1.7, 13.1)↧ | ⨁⨁⨁◯Moderate |
| high Potassium vs isometric exercise | E vs F | - | - | 6.51 (1.14, 11.98)* | ⨁⨁⨁◯Moderate | 6.51 (1.14, 11.98)↧ | ⨁⨁⨁◯Moderate |
| acupuncture vs high Potassium | A vs E | - | - | -5.43 (-13.28, 2.43)*⇟ | ⨁⨁⨁◯Moderate | -5.43 (-13.28, 2.43)↧ | ⨁⨁◯◯Low |
| aerobic exercise vs high Potassium | B vs E | - | - | -5.05 (-10.15, 0.07)*⇟ | ⨁⨁⨁◯Moderate | -5.05 (-10.15, 0.07)↧ | ⨁⨁◯◯Low |
| combination exercise vs high Potassium | C vs E | - | - | -9.37 (-15.87, -2.87)* | ⨁⨁⨁◯Moderate | -9.37 (-15.87, -2.87)↧ | ⨁⨁⨁◯Moderate |
| DASH vs high Potassium | D vs E | - | - | 1.12 (-5.35, 7.1)*⇟ | ⨁⨁⨁◯Moderate | 1.12 (-5.35, 7.1)↧ | ⨁⨁◯◯Low |
| acupuncture vs DASH | A vs D | - | - | -6.56 (-14.41, 1.76)*⇟ | ⨁⨁⨁◯Moderate | -6.56 (-14.41, 1.76)↧ | ⨁⨁◯◯Low |
| aerobic exercise vs DASH | B vs D | - | - | -6.16 (-11.37, -0.52)* | ⨁⨁⨁◯Moderate | -6.16 (-11.37, -0.52)↧ | ⨁⨁⨁◯Moderate |
| combination exercise vs DASH | C vs D | - | - | -10.48 (-17.01, -3.51)* | ⨁⨁⨁◯Moderate | -10.48 (-17.01, -3.51)↧ | ⨁⨁⨁◯Moderate |
| acupuncture vs combination exercise | A vs C | - | - | 3.9 (-4.35, 12.28)*⇟ | ⨁⨁⨁◯Moderate | 3.9 (-4.35, 12.28)↧ | ⨁⨁◯◯Low |
| aerobic exercise vs combination exercise | B vs C | - | - | 4.32 (-1.31, 9.97)*⇟ | ⨁⨁⨁◯Moderate | 4.32 (-1.31, 9.97)↧ | ⨁⨁◯◯Low |
| acupuncture vs aerobic exercise | A vs B | - | - | -0.4 (-7.67, 6.93)*⇟ | ⨁⨁⨁◯Moderate | -0.4 (-7.67, 6.93)↧ | ⨁⨁◯◯Low |
| ↑↑ Not assessed,This treatment does not included in the Network; † Serious risk of bias; * Serious Indirectness; ⇟ Imprecision; ↥ Direct evidence contributes more; ↧Indirect evidence contributes more; MD: Mean difference; CI: Confidence interval; § Heterogeneity | | | | | | | |

**Table 2 Certainty of the evidence in the DBP reduction of Interventions**

| **Name** | **ID** | **Direct evidence**  **MD (95%CI)** | **Evidence level** | **Indirect evidence**  **MD (95%CI)** | **Evidence level** | **Network evidence**  **MD (95%CI)** | **Evidence level** |
| --- | --- | --- | --- | --- | --- | --- | --- |
| acupuncture vs usual care | A vs O | -7.08 (-9.95, -4.21)* | ⨁⨁⨁◯Moderate | ↑↑ | - | -6.48 (-11.39, -1.42)↥ | ⨁⨁⨁◯Moderate |
| aerobic exercise vs usual care | B vs O | -2.7 (-5.2, -0.16)*§ | ⨁⨁◯◯Low | -7.1 (-15, 0.39)*⇟ | ⨁⨁⨁◯Moderate | -3.12 (-5.51, -0.73)↥ | ⨁⨁◯◯Low |
| combination exercise vs usual care | C vs O | -3.89 (-6.09, -1.68)* | ⨁⨁⨁◯Moderate | -4.67 (-8.56, -0.79)* | ⨁⨁⨁◯Moderate | -4.67 (-8.56, -0.79)↥ | ⨁⨁⨁◯Moderate |
| DASH vs usual care | D vs O | 0.43 (-3.6, 4.4)⇟§ | ⨁⨁⨁◯Moderate | -3.5 (-10, 3.1)*⇟ | ⨁⨁⨁◯Moderate | -0.62 (-4.21, 2.7)↥ | ⨁⨁⨁◯Moderate |
| high Potassium vs usual care | E vs O | -0.41 (-1.23, 0.40)*⇟ | ⨁⨁⨁◯Moderate | -0.91 (-4.39, 2.47)*⇟ | ⨁⨁⨁◯Moderate | -0.91 (-4.39, 2.47)↥ | ⨁⨁◯◯Low |
| isometric exercise vs usual care | F vs O | -3.8 (-8.2, 0.46)*⇟§ | ⨁⨁◯◯Low | -3.4 (-7.8, 1.1)*⇟ | ⨁⨁⨁◯Moderate | -3.88 (-6.74, -0.95)↥ | ⨁◯◯◯Very low |
| Lifestyle vs usual care(bias) | G vs O | -3.2 (-5.5, -0.79)†§ | ⨁⨁⨁◯Moderate | -1 (-4.9, 2.8)† *⇟ | ⨁⨁◯◯Low | -2.56 (-4.56, -0.58)↥ | ⨁⨁⨁◯Moderate |
| meditation vs usual care | H vs O | -5.5 (-9.9, -1.2)* | ⨁⨁⨁◯Moderate | -6.5 (-13, 0.15)*⇟ | ⨁⨁⨁◯Moderate | -5.81 (-9.41, -2.19)↥ | ⨁⨁⨁◯Moderate |
| normal exercise vs usual care | I vs O | -2.8 (-6.2, 0.53)*⇟§ | ⨁⨁◯◯Low | -2.5 (-10, 5)*⇟ | ⨁⨁⨁◯Moderate | -3.32 (-6.05, -0.59)↥ | ⨁◯◯◯Very low |
| reduced alcohol vs usual care | J vs O | -2.03 (-2.70, -1.37)* | ⨁⨁⨁◯Moderate | ↑↑ | - | -2.43 (-5.89, 0.93)↥ | ⨁⨁⨁◯Moderate |
| resistance exercise vs usual care | K vs O | -3.06 (-5.36, -0.77)*§ | ⨁⨁◯◯Low | -4.02 (-8.36, 0.18)*⇟ | ⨁⨁⨁◯Moderate | -4.02 (-8.36, 0.18)↥ | ⨁⨁◯◯Low |
| salt restriction vs usual care | L vs O | -0.27 (-0.56, 0.02)*⇟§ | ⨁⨁◯◯Low | -1.85 (-4.1, 0.25)*⇟ | ⨁⨁⨁◯Moderate | -1.85 (-4.1, 0.25)↥ | ⨁◯◯◯Very low |
| weight loss vs usual care | M vs O | -2.72 (-3.34, -2.10)* | ⨁⨁⨁◯Moderate | -3.16 (-6.14, -0.29)* | ⨁⨁⨁◯Moderate | -3.16 (-6.14, -0.29)↥ | ⨁⨁⨁◯Moderate |
| yoga vs usual care | N vs O | -5 (-9, -1.1)*§ | ⨁⨁◯◯Low | -3.5 (-9.1, 1.9)*⇟ | ⨁⨁⨁◯Moderate | -3.73 (-6.81, -0.59)↥ | ⨁⨁◯◯Low |
| acupuncture vs yoga | A vs N | - | - | -2.75 (-8.6, 3.14)*⇟ | ⨁⨁⨁◯Moderate | -2.75 (-8.6, 3.14)↧ | ⨁⨁◯◯Low |
| aerobic exercise vs yoga | B vs N | - | - | 0.62 (-3.3, 4.46)*⇟ | ⨁⨁⨁◯Moderate | 0.62 (-3.3, 4.46)↧ | ⨁⨁◯◯Low |
| combination exercise vs yoga | C vs N | - | - | -0.93 (-5.89, 3.93)*⇟ | ⨁⨁⨁◯Moderate | -0.93 (-5.89, 3.93)↧ | ⨁⨁◯◯Low |
| DASH vs yoga | D vs N | - | - | 3.1 (-1.58, 7.52)*⇟ | ⨁⨁⨁◯Moderate | 3.1 (-1.58, 7.52)↧ | ⨁⨁◯◯Low |
| high Potassium vs yoga | E vs N | - | - | 2.81 (-1.8, 7.32)*⇟ | ⨁⨁⨁◯Moderate | 2.81 (-1.8, 7.32)↧ | ⨁⨁◯◯Low |
| isometric exercise vs yoga | F vs N | - | - | -0.14 (-4.26, 3.96)*⇟ | ⨁⨁⨁◯Moderate | -0.14 (-4.26, 3.96)↧ | ⨁⨁◯◯Low |
| Lifestyle vs yoga | G vs N | 0.98 (-4.4, 6.4)*⇟ | ⨁⨁⨁◯Moderate | 1.2 (-3.2, 5.5)*⇟ | ⨁⨁⨁◯Moderate | 1.17 (-2.17, 4.44)↧ | ⨁⨁◯◯Low |
| meditation vs yoga | H vs N | - | - | -2.08 (-6.76, 2.54)*⇟ | ⨁⨁⨁◯Moderate | -2.08 (-6.76, 2.54)↧ | ⨁⨁◯◯Low |
| normal exercise vs yoga | I vs N | -3.4 (-9.2, 2.5)*⇟ | ⨁⨁⨁◯Moderate | 3.1 (-1.9, 8.1)*⇟ | ⨁⨁⨁◯Moderate | 0.42 (-3.41, 4.2)↧ | ⨁⨁◯◯Low |
| reduced alcohol vs yoga | J vs N | - | - | 1.3 (-3.39, 5.85)*⇟ | ⨁⨁⨁◯Moderate | 1.3 (-3.39, 5.85)↧ | ⨁⨁◯◯Low |
| resistance exercise vs yoga | K vs N | - | - | -0.3 (-5.65, 4.89)*⇟ | ⨁⨁⨁◯Moderate | -0.3 (-5.65, 4.89)↧ | ⨁⨁◯◯Low |
| salt restriction vs yoga | L vs N | -1.1 (-7.2, 5)*⇟ | ⨁⨁⨁◯Moderate | 3.7 (-0.94, 8.1)*⇟ | ⨁⨁⨁◯Moderate | 1.88 (-1.8, 5.39)↧ | ⨁⨁◯◯Low |
| weight loss vs yoga | M vs N | - | - | 0.56 (-3.74, 4.71)*⇟ | ⨁⨁⨁◯Moderate | 0.56 (-3.74, 4.71)↧ | ⨁⨁◯◯Low |
| acupuncture vs weight loss | A vs M | - | - | -3.32 (-9, 2.6)*⇟ | ⨁⨁⨁◯Moderate | -3.32 (-9, 2.6)↧ | ⨁⨁◯◯Low |
| aerobic exercise vs weight loss | B vs M | - | - | 0.05 (-3.7, 3.9)*⇟ | ⨁⨁⨁◯Moderate | 0.05 (-3.7, 3.9)↧ | ⨁⨁◯◯Low |
| combination exercise vs weight loss | C vs M | - | - | -1.49 (-6.33, 3.38)*⇟ | ⨁⨁⨁◯Moderate | -1.49 (-6.33, 3.38)↧ | ⨁⨁◯◯Low |
| DASH vs weight loss | D vs M | - | - | 2.55 (-2.08, 6.99)*⇟ | ⨁⨁⨁◯Moderate | 2.55 (-2.08, 6.99)↧ | ⨁⨁◯◯Low |
| high Potassium vs weight loss | E vs M | - | - | 2.25 (-2.11, 6.67)*⇟ | ⨁⨁⨁◯Moderate | 2.25 (-2.11, 6.67)↧ | ⨁⨁◯◯Low |
| isometric exercise vs weight loss | F vs M | - | - | -0.71 (-4.76, 3.47)*⇟ | ⨁⨁⨁◯Moderate | -0.71 (-4.76, 3.47)↧ | ⨁⨁◯◯Low |
| Lifestyle vs weight loss | G vs M | - | - | 0.62 (-2.84, 4.2)*⇟ | ⨁⨁⨁◯Moderate | 0.62 (-2.84, 4.2)↧ | ⨁⨁◯◯Low |
| meditation vs weight loss | H vs M | - | - | -2.65 (-7.22, 2.06)*⇟ | ⨁⨁⨁◯Moderate | -2.65 (-7.22, 2.06)↧ | ⨁⨁◯◯Low |
| normal exercise vs weight loss | I vs M | - | - | -0.14 (-4.03, 3.82)*⇟ | ⨁⨁⨁◯Moderate | -0.14 (-4.03, 3.82)↧ | ⨁⨁◯◯Low |
| reduced alcohol vs weight loss | J vs M | - | - | 0.73 (-3.74, 5.26)*⇟ | ⨁⨁⨁◯Moderate | 0.73 (-3.74, 5.26)↧ | ⨁⨁◯◯Low |
| resistance exercise vs weight loss | K vs M | - | - | -0.86 (-6.06, 4.32)*⇟ | ⨁⨁⨁◯Moderate | -0.86 (-6.06, 4.32)↧ | ⨁⨁◯◯Low |
| salt restriction vs weight loss | L vs M | 1.6 (-2.6, 5.8)*⇟ | ⨁⨁⨁◯Moderate | 0.56 (-5.6, 6.6)*⇟ | ⨁⨁⨁◯Moderate | 1.31 (-1.89, 4.49)↥ | ⨁⨁◯◯Low |
| acupuncture vs salt restriction | A vs L | - | - | -4.63 (-9.94, 0.91)*⇟ | ⨁⨁⨁◯Moderate | -4.63 (-9.94, 0.91)↧ | ⨁⨁◯◯Low |
| aerobic exercise vs salt restriction | B vs L | - | - | -1.27 (-4.43, 2.03)*⇟ | ⨁⨁⨁◯Moderate | -1.27 (-4.43, 2.03)↧ | ⨁⨁◯◯Low |
| combination exercise vs salt restriction | C vs L | - | - | -2.8 (-7.15, 1.62)*⇟ | ⨁⨁⨁◯Moderate | -2.8 (-7.15, 1.62)↧ | ⨁⨁◯◯Low |
| DASH vs salt restriction | D vs L | - | - | 1.22 (-2.89, 5.21)*⇟ | ⨁⨁⨁◯Moderate | 1.22 (-2.89, 5.21)↧ | ⨁⨁◯◯Low |
| high Potassium vs salt restriction | E vs L | -0.3 (-5.8, 5.1)*⇟ | ⨁⨁⨁◯Moderate | 2 (-3.8, 7.9)*⇟ | ⨁⨁⨁◯Moderate | 0.93 (-2.72, 4.68)↥ | ⨁⨁◯◯Low |
| isometric exercise vs salt restriction | F vs L | - | - | -2.02 (-5.49, 1.62)*⇟ | ⨁⨁⨁◯Moderate | -2.02 (-5.49, 1.62)↧ | ⨁⨁◯◯Low |
| Lifestyle vs salt restriction | G vs L | - | - | -0.72 (-3.55, 2.27)*⇟ | ⨁⨁⨁◯Moderate | -0.72 (-3.55, 2.27)↧ | ⨁⨁◯◯Low |
| meditation vs salt restriction | H vs L | - | - | -3.96 (-8.06, 0.29)*⇟ | ⨁⨁⨁◯Moderate | -3.96 (-8.06, 0.29)↧ | ⨁⨁◯◯Low |
| normal exercise vs salt restriction | I vs L | -2.3 (-8, 3.5)*⇟ | ⨁⨁⨁◯Moderate | -0.53 (-4.5, 3.5)*⇟ | ⨁⨁⨁◯Moderate | -1.46 (-4.69, 1.9)↧ | ⨁⨁◯◯Low |
| reduced alcohol vs salt restriction | J vs L | - | - | -0.58 (-4.58, 3.5)*⇟ | ⨁⨁⨁◯Moderate | -0.58 (-4.58, 3.5)↧ | ⨁⨁◯◯Low |
| resistance exercise vs salt restriction | K vs L | - | - | -2.18 (-6.95, 2.59)*⇟ | ⨁⨁⨁◯Moderate | -2.18 (-6.95, 2.59)↧ | ⨁⨁◯◯Low |
| acupuncture vs resistance exercise | A vs K | - | - | -2.45 (-8.95, 4.29)*⇟ | ⨁⨁⨁◯Moderate | -2.45 (-8.95, 4.29)↧ | ⨁⨁◯◯Low |
| aerobic exercise vs resistance exercise | B vs K | - | - | 0.9 (-3.88, 5.81)*⇟ | ⨁⨁⨁◯Moderate | 0.9 (-3.88, 5.81)↧ | ⨁⨁◯◯Low |
| combination exercise vs resistance exercise | C vs K | 1.1 (-6.2, 8.1)*⇟ | ⨁⨁⨁◯Moderate | -2.5 (-10, 5.1)*⇟ | ⨁⨁⨁◯Moderate | -0.63 (-5.69, 4.51)↥ | ⨁⨁◯◯Low |
| DASH vs resistance exercise | D vs K | - | - | 3.41 (-2.13, 8.82)*⇟ | ⨁⨁⨁◯Moderate | 3.41 (-2.13, 8.82)↧ | ⨁⨁◯◯Low |
| high Potassium vs resistance exercise | E vs K | - | - | 3.12 (-2.27, 8.67)*⇟ | ⨁⨁⨁◯Moderate | 3.12 (-2.27, 8.67)↧ | ⨁⨁◯◯Low |
| isometric exercise vs resistance exercise | F vs K | - | - | 0.15 (-4.86, 5.31)*⇟ | ⨁⨁⨁◯Moderate | 0.15 (-4.86, 5.31)↧ | ⨁⨁◯◯Low |
| Lifestyle vs resistance exercise | G vs K | - | - | 1.48 (-3.18, 6.2)*⇟ | ⨁⨁⨁◯Moderate | 1.48 (-3.18, 6.2)↧ | ⨁⨁◯◯Low |
| meditation vs resistance exercise | H vs K | - | - | -1.78 (-7.28, 3.92)*⇟ | ⨁⨁⨁◯Moderate | -1.78 (-7.28, 3.92)↧ | ⨁⨁◯◯Low |
| normal exercise vs resistance exercise | I vs K | - | - | 0.73 (-4.19, 5.73)*⇟ | ⨁⨁⨁◯Moderate | 0.73 (-4.19, 5.73)↧ | ⨁⨁◯◯Low |
| reduced alcohol vs resistance exercise | J vs K | - | - | 1.61 (-3.85, 7.11)*⇟ | ⨁⨁⨁◯Moderate | 1.61 (-3.85, 7.11)↧ | ⨁⨁◯◯Low |
| acupuncture vs reduced alcohol | A vs J | - | - | -4.04 (-9.99, 2.09)*⇟ | ⨁⨁⨁◯Moderate | -4.04 (-9.99, 2.09)↧ | ⨁⨁◯◯Low |
| aerobic exercise vs reduced alcohol | B vs J | - | - | -0.69 (-4.81, 3.51)*⇟ | ⨁⨁⨁◯Moderate | -0.69 (-4.81, 3.51)↧ | ⨁⨁◯◯Low |
| combination exercise vs reduced alcohol | C vs J | - | - | -2.24 (-7.38, 2.95)*⇟ | ⨁⨁⨁◯Moderate | -2.24 (-7.38, 2.95)↧ | ⨁⨁◯◯Low |
| DASH vs reduced alcohol | D vs J | - | - | 1.8 (-3.1, 6.57)*⇟ | ⨁⨁⨁◯Moderate | 1.8 (-3.1, 6.57)↧ | ⨁⨁◯◯Low |
| high Potassium vs reduced alcohol | E vs J | - | - | 1.51 (-3.31, 6.32)*⇟ | ⨁⨁⨁◯Moderate | 1.51 (-3.31, 6.32)↧ | ⨁⨁◯◯Low |
| isometric exercise vs reduced alcohol | F vs J | - | - | -1.45 (-5.86, 3.09)*⇟ | ⨁⨁⨁◯Moderate | -1.45 (-5.86, 3.09)↧ | ⨁⨁◯◯Low |
| Lifestyle vs reduced alcohol | G vs J | - | - | -0.13 (-4.03, 3.86)*⇟ | ⨁⨁⨁◯Moderate | -0.13 (-4.03, 3.86)↧ | ⨁⨁◯◯Low |
| meditation vs reduced alcohol | H vs J | - | - | -3.37 (-8.3, 1.63)*⇟ | ⨁⨁⨁◯Moderate | -3.37 (-8.3, 1.63)↧ | ⨁⨁◯◯Low |
| normal exercise vs reduced alcohol | I vs J | - | - | -0.88 (-5.23, 3.49)*⇟ | ⨁⨁⨁◯Moderate | -0.88 (-5.23, 3.49)↧ | ⨁⨁◯◯Low |
| acupuncture vs normal exercise | A vs I | - | - | -3.18 (-8.77, 2.6)*⇟ | ⨁⨁⨁◯Moderate | -3.18 (-8.77, 2.6)↧ | ⨁⨁◯◯Low |
| aerobic exercise vs normal exercise | B vs I | - | - | 0.2 (-3.36, 3.8)*⇟ | ⨁⨁⨁◯Moderate | 0.2 (-3.36, 3.8)↧ | ⨁⨁◯◯Low |
| combination exercise vs normal exercise | C vs I | -1.9 (-7.7, 3.7)*⇟ | ⨁⨁⨁◯Moderate | -1.2 (-8.7, 6.1)*⇟ | ⨁⨁⨁◯Moderate | -1.36 (-5.64, 2.96)↥ | ⨁⨁◯◯Low |
| DASH vs normal exercise | D vs I | - | - | 2.68 (-1.78, 6.97)*⇟ | ⨁⨁⨁◯Moderate | 2.68 (-1.78, 6.97)↧ | ⨁⨁◯◯Low |
| high Potassium vs normal exercise | E vs I | - | - | 2.4 (-1.91, 6.67)*⇟ | ⨁⨁⨁◯Moderate | 2.4 (-1.91, 6.67)↧ | ⨁⨁◯◯Low |
| isometric exercise vs normal exercise | F vs I | -0.57 (-6.3, 5.2)*⇟ | ⨁⨁⨁◯Moderate | -0.58 (-5.4, 4.4)*⇟ | ⨁⨁⨁◯Moderate | -0.55 (-4.18, 3.1)↧ | ⨁⨁◯◯Low |
| Lifestyle vs normal exercise | G vs I | - | - | 0.75 (-2.51, 4.02)*⇟ | ⨁⨁⨁◯Moderate | 0.75 (-2.51, 4.02)↧ | ⨁⨁◯◯Low |
| meditation vs normal exercise | H vs I | -2.9 (-9, 3)*⇟ | ⨁⨁⨁◯Moderate | -2.1 (-7.4, 3.2)*⇟ | ⨁⨁⨁◯Moderate | -2.5 (-6.4, 1.45)↧ | ⨁⨁◯◯Low |
| acupuncture vs meditation | A vs H | - | - | -0.7 (-6.74, 5.53)*⇟ | ⨁⨁⨁◯Moderate | -0.7 (-6.74, 5.53)↧ | ⨁⨁◯◯Low |
| aerobic exercise vs meditation | B vs H | - | - | 2.68 (-1.63, 7.02)*⇟ | ⨁⨁⨁◯Moderate | 2.68 (-1.63, 7.02)↧ | ⨁⨁◯◯Low |
| combination exercise vs meditation | C vs H | - | - | 1.13 (-3.98, 6.28)*⇟ | ⨁⨁⨁◯Moderate | 1.13 (-3.98, 6.28)↧ | ⨁⨁◯◯Low |
| DASH vs meditation | D vs H | - | - | 5.18 (0.03, 10.06)* | ⨁⨁⨁◯Moderate | 5.18 (0.03, 10.06)↧ | ⨁⨁⨁◯Moderate |
| high Potassium vs meditation | E vs H | - | - | 4.89 (-0.11, 9.81)*⇟ | ⨁⨁⨁◯Moderate | 4.89 (-0.11, 9.81)↧ | ⨁⨁◯◯Low |
| isometric exercise vs meditation | F vs H | - | - | 1.94 (-2.59, 6.49)*⇟ | ⨁⨁⨁◯Moderate | 1.94 (-2.59, 6.49)↧ | ⨁⨁◯◯Low |
| Lifestyle vs meditation | G vs H | - | - | 3.26 (-0.87, 7.32)*⇟ | ⨁⨁⨁◯Moderate | 3.26 (-0.87, 7.32)↧ | ⨁⨁◯◯Low |
| acupuncture vs Lifestyle | A vs G | - | - | -3.93 (-9.21, 1.48)*⇟ | ⨁⨁⨁◯Moderate | -3.93 (-9.21, 1.48)↧ | ⨁⨁◯◯Low |
| aerobic exercise vs Lifestyle | B vs G | - | - | -0.56 (-3.63, 2.5)*⇟ | ⨁⨁⨁◯Moderate | -0.56 (-3.63, 2.5)↧ | ⨁⨁◯◯Low |
| combination exercise vs Lifestyle | C vs G | - | - | -2.1 (-6.41, 2.14)*⇟ | ⨁⨁⨁◯Moderate | -2.1 (-6.41, 2.14)↧ | ⨁⨁◯◯Low |
| DASH vs Lifestyle | D vs G | -0.84 (-7.2, 5.5)*⇟ | ⨁⨁⨁◯Moderate | 3.3 (-1.3, 7.7)*⇟ | ⨁⨁⨁◯Moderate | 1.93 (-1.84, 5.54)↧ | ⨁⨁◯◯Low |
| high Potassium vs Lifestyle | E vs G | - | - | 1.65 (-2.37, 5.56)*⇟ | ⨁⨁⨁◯Moderate | 1.65 (-2.37, 5.56)↧ | ⨁⨁◯◯Low |
| isometric exercise vs Lifestyle | F vs G | -2.5 (-7.7, 2.6)*⇟ | ⨁⨁⨁◯Moderate | -0.5 (-4.5, 3.6)*⇟ | ⨁⨁⨁◯Moderate | -1.32 (-4.42, 1.83)↧ | ⨁⨁◯◯Low |
| acupuncture vs isometric exercise | A vs F | - | - | -2.6 (-8.37, 3.18)*⇟ | ⨁⨁⨁◯Moderate | -2.6 (-8.37, 3.18)↧ | ⨁⨁◯◯Low |
| aerobic exercise vs isometric exercise | B vs F | -2.6 (-9.5, 4.1)*⇟ | ⨁⨁⨁◯Moderate | 1.9 (-2.1, 5.9)*⇟ | ⨁⨁⨁◯Moderate | 0.74 (-2.72, 4.22)↧ | ⨁⨁◯◯Low |
| combination exercise vs isometric exercise | C vs F | -1.3 (-7.2, 4.7)*⇟ | ⨁⨁⨁◯Moderate | -0.66 (-8.4, 6.8)*⇟ | ⨁⨁⨁◯Moderate | -0.81 (-5.24, 3.6)↥ | ⨁⨁◯◯Low |
| DASH vs isometric exercise | D vs F | - | - | 3.24 (-1.28, 7.56)*⇟ | ⨁⨁⨁◯Moderate | 3.24 (-1.28, 7.56)↧ | ⨁⨁◯◯Low |
| high Potassium vs isometric exercise | E vs F | - | - | 2.96 (-1.59, 7.4)*⇟ | ⨁⨁⨁◯Moderate | 2.96 (-1.59, 7.4)↧ | ⨁⨁◯◯Low |
| acupuncture vs high Potassium | A vs E | - | - | -5.56 (-11.48, 0.61)*⇟ | ⨁⨁⨁◯Moderate | -5.56 (-11.48, 0.61)↧ | ⨁⨁◯◯Low |
| aerobic exercise vs high Potassium | B vs E | - | - | -2.22 (-6.34, 2.02)*⇟ | ⨁⨁⨁◯Moderate | -2.22 (-6.34, 2.02)↧ | ⨁⨁◯◯Low |
| combination exercise vs high Potassium | C vs E | - | - | -3.76 (-8.92, 1.35)*⇟ | ⨁⨁⨁◯Moderate | -3.76 (-8.92, 1.35)↧ | ⨁⨁◯◯Low |
| DASH vs high Potassium | D vs E | - | - | 0.29 (-4.65, 5.02)*⇟ | ⨁⨁⨁◯Moderate | 0.29 (-4.65, 5.02)↧ | ⨁⨁◯◯Low |
| acupuncture vs DASH | A vs D | - | - | -5.85 (-11.77, 0.39)*⇟ | ⨁⨁⨁◯Moderate | -5.85 (-11.77, 0.39)↧ | ⨁⨁◯◯Low |
| aerobic exercise vs DASH | B vs D | - | - | -2.5 (-6.58, 1.82)*⇟ | ⨁⨁⨁◯Moderate | -2.5 (-6.58, 1.82)↧ | ⨁⨁◯◯Low |
| combination exercise vs DASH | C vs D | - | - | -4.03 (-9.1, 1.22)*⇟ | ⨁⨁⨁◯Moderate | -4.03 (-9.1, 1.22)↧ | ⨁⨁◯◯Low |
| acupuncture vs combination exercise | A vs C | - | - | -1.84 (-8.11, 4.61)*⇟ | ⨁⨁⨁◯Moderate | -1.84 (-8.11, 4.61)↧ | ⨁⨁◯◯Low |
| aerobic exercise vs combination exercise | B vs C | - | - | 1.53 (-2.95, 6.01)*⇟ | ⨁⨁⨁◯Moderate | 1.53 (-2.95, 6.01)↧ | ⨁⨁◯◯Low |
| acupuncture vs aerobic exercise | A vs B | - | - | -3.36 (-8.81, 2.23)*⇟ | ⨁⨁⨁◯Moderate | -3.36 (-8.81, 2.23)↧ | ⨁⨁◯◯Low |
| ↑↑ Not assessed,This treatment does not included in the Network; † Serious risk of bias; * Serious Indirectness; ⇟ Imprecision; ↥ Direct evidence contributes more; ↧Indirect evidence contributes more; MD: Mean difference; CI: Confidence interval; § Heterogeneity | | | | | | | |

**Table 3 Certainty of the evidence in the SBP reduction of Intervention groups**

| **Name** | **ID** | **Direct evidence**  **MD (95%CI)** | **Evidence level** | **Indirect evidence**  **MD (95%CI)** | **Evidence level** | **Network evidence**  **MD (95%CI)** | **Evidence level** |
| --- | --- | --- | --- | --- | --- | --- | --- |
| Relaxation vs Strengthen Exercise | A vs E | 2.2 (-3.5, 7.8)* | ⨁⨁⨁◯  Moderate | 0.38 (-3.6, 4.5)*⇟ | ⨁⨁⨁◯  Moderate | 1.05 (-2.09, 4.28)↧ | ⨁⨁◯◯  Low |
| Usual Care vs Strengthen Exercise | B vs E | 5.8 (3.3, 8.2)*§ | ⨁⨁◯◯  Low | 6.9 (1.4, 12)* | ⨁⨁⨁◯  Moderate | 6.02 (3.87, 8.16)↥ | ⨁⨁◯◯  Low |
| Lifestyle vs Strengthen Exercise | C vs E | 3.4 (-3.4, 10)* | ⨁⨁⨁◯  Moderate | 2.3 (-0.88, 5.5)*⇟ | ⨁⨁⨁◯  Moderate | 2.51 (-0.33, 5.3)↧ | ⨁⨁◯◯  Low |
| Dietary vs Strengthen Exercise | D vs E | 2.7 (-5.1, 10)* | ⨁⨁⨁◯  Moderate | 3.6 (0.23, 6.7)* | ⨁⨁⨁◯  Moderate | 3.47 (0.53, 6.36)↧ | ⨁⨁⨁◯  Moderate |
| Relaxation vs Dietary | A vs D | 0.62 (-7.8, 8.9)* | ⨁⨁⨁◯  Moderate | -3 (-6.7, 0.87)*⇟ | ⨁⨁⨁◯  Moderate | -2.43 (-5.71, 1.07)↧ | ⨁⨁◯◯  Low |
| Usual Care vs Dietary | B vs D | 2.4 (0.14, 4.9)*§ | ⨁⨁◯◯  Low | 3.5 (-6.8, 14)* | ⨁⨁⨁◯  Moderate | 2.54 (0.49, 4.73)↥ | ⨁⨁◯◯  Low |
| Lifestyle vs Dietary | C vs D | -1.2 (-6.1, 3.7)* | ⨁⨁⨁◯  Moderate | -0.8 (-4.3, 2.9)*⇟ | ⨁⨁⨁◯  Moderate | -0.97 (-3.67, 1.79)↧ | ⨁⨁◯◯  Low |
| Relaxation vs Lifestyle | A vs C | -2 (-8.9, 4.9)* | ⨁⨁⨁◯  Moderate | -1.2 (-4.9, 2.6)*⇟ | ⨁⨁⨁◯  Moderate | -1.48 (-4.61, 1.83)↧ | ⨁⨁◯◯  Low |
| Usual Care vs Lifestyle | B vs C | 3.8 (1.2, 6.4)§ | ⨁⨁⨁◯  Moderate | 2.6 (-2.2, 7.3) | ⨁⨁⨁⨁  High | 3.5 (1.41, 5.64)↥ | ⨁⨁⨁◯  Moderate |
| Relaxation vs Usual Care | A vs B | -5 (-8.3, -1.5)*§ | ⨁⨁◯◯  Low | -5.1 (-11, 0.33)* | ⨁⨁⨁◯  Moderate | -4.97 (-7.66, -2.15)↥ | ⨁⨁◯◯  Low |
| * Serious Indirectness; ⇟ Imprecision; ↥ Direct evidence contributes more; ↧Indirect evidence contributes more; MD: Mean difference; CI: Confidence interval; § Heterogeneity | | | | | | | |

**Table 4 Certainty of the evidence in the DBP reduction of Intervention groups**

| **Name** | **ID** | **Direct evidence**  **MD (95%CI)** | **Evidence level** | **Indirect evidence**  **MD (95%CI)** | **Evidence level** | **Network evidence**  **MD (95%CI)** | **Evidence level** |
| --- | --- | --- | --- | --- | --- | --- | --- |
| Relaxation vs Strengthen Exercise | A vs E | 0.33 (-3.8, 4.4)*§ | ⨁⨁◯◯  Low | -2.5 (-5.5, 0.52)*⇟ | ⨁⨁⨁◯  Moderate | -1.52 (-3.94, 0.88)↧ | ⨁⨁◯◯  Low |
| Usual Care vs Strengthen Exercise | B vs E | 3.2 (1.3, 5)*§ | ⨁⨁◯◯  Low | 4.2 (0.15, 8.2)* | ⨁⨁⨁◯  Moderate | 3.48 (1.82, 5.09)↥ | ⨁⨁◯◯  Low |
| Lifestyle vs Strengthen Exercise | C vs E | 2.5 (-2.3, 7.3)* | ⨁⨁⨁◯  Moderate | 0.15 (-2.2, 2.5)*⇟ | ⨁⨁⨁◯  Moderate | 0.61 (-1.53, 2.68)↧ | ⨁⨁◯◯  Low |
| Dietary vs Strengthen Exercise | D vs E | 2.3 (-3.2, 7.8)* | ⨁⨁⨁◯  Moderate | 1.6 (-0.73, 3.8)*⇟ | ⨁⨁⨁◯  Moderate | 1.74 (-0.49, 3.87)↧ | ⨁⨁◯◯  Low |
| Relaxation vs Dietary | A vs D | 1.2 (-4.6, 6.9)* | ⨁⨁⨁◯  Moderate | -4.3 (-6.9, -1.5)* | ⨁⨁⨁◯  Moderate | -3.25 (-5.68, -0.73)↧ | ⨁⨁⨁◯  Moderate |
| Usual Care vs Dietary | B vs D | 1.7 (-0.056, 3.5)*⇟§ | ⨁⨁◯◯  Low | 3.7 (-3, 11)* | ⨁⨁⨁◯  Moderate | 1.73 (0.23, 3.34)↥ | ⨁◯◯◯  Very low |
| Lifestyle vs Dietary | C vs D | -0.96 (-4.4, 2.5)* | ⨁⨁⨁◯  Moderate | -1.2 (-3.8, 1.5)*⇟ | ⨁⨁⨁◯  Moderate | -1.14 (-3.07, 0.91)↧ | ⨁⨁◯◯  Low |
| Relaxation vs Lifestyle | A vs C | -0.97 (-6.1, 4.1)* | ⨁⨁⨁◯  Moderate | -2.4 (-5.1, 0.3)*⇟ | ⨁⨁⨁◯  Moderate | -2.12 (-4.49, 0.24)↧ | ⨁⨁◯◯  Low |
| Usual Care vs Lifestyle | B vs C | 3.1 (1.2, 5)§ | ⨁⨁⨁◯  Moderate | 2 (-1.5, 5.4) | ⨁⨁⨁⨁  High | 2.86 (1.34, 4.43)↥ | ⨁⨁⨁◯  Moderate |
| Relaxation vs Usual Care | A vs B | -5.6 (-8, -3.2)* | ⨁⨁⨁◯  Moderate | -4.8 (-8.6, -0.96)* | ⨁⨁⨁◯  Moderate | -4.99 (-7.03, -2.96)↥ | ⨁⨁⨁◯  Moderate |
| * Serious Indirectness; ⇟ Imprecision; ↥ Direct evidence contributes more; ↧Indirect evidence contributes more; MD: Mean difference; CI: Confidence interval; § Heterogeneity | | | | | | | |
